# Single‐cell‐based non‐invasive screening for fetal pathogenic microimbalances using maternal blood: comparison with invasive prenatal diagnosis

**DOI:** 10.1002/uog.70201

**Published:** 2026-03-25

**Authors:** T. Stampalija, C. Forcato, F. R. Grati, P. Volpe, V. De Robertis, C. Izzi, E. Bertucci, I. Fabietti, A. Novelli, S. Ornaghi, L. Pasquini, E. Bevilacqua, D. Paladini, T. Ghi, D. Lattuada, M. Dori, D. Mercatelli, A. Dal Molin, G. Buson, C. Bolognesi, A. Doffini, T. J. Musci, E. Ferrazzi, Collaborators, Emilia Dora Giovannone, Chiara Mangano, Chiara Maranta, Camilla Amadesi, Antonio Brocco, Arianna Casadei, Melissa Garrì, Rebecca Maiocchi, Ilaria Molinaro, Elisa Ortolan, Angela Piano, Maria Chiara Iannitiello, Davide Lisi, Agnese Feresin, Paolo Gasparini, Camilla Fregona, Georgios Rembouskos, Franco Edoardo Odicino, Marino Signorelli, Alessandra Sponzilli, Fabio Facchinetti, Chiara Vassallo, Elena Nicastri, Maria Grazia Di Gregorio, Valeria Orlando, Sabrina Cozzolino, Maria Verderio, Giulia Masini, Chiara Franchi, Francesco Danilo Tiziano, Domizia Pasquetti, Giulia Biancotto, Francesca Della Sala, Nicola Volpe, Andrea Dall'Asta, Rosamaria Silipigni, Ilaria Catusi

**Affiliations:** ^1^ Unit of Fetal Medicine and Prenatal Diagnosis Institute for Maternal and Child Health IRCCS Burlo Garofolo Trieste Italy; ^2^ Department of Medical, Surgical and Health Sciences University of Trieste Trieste Italy; ^3^ Reproductive Precision Medicine Unit, Menarini Silicon Biosystems Castel Maggiore (Bologna) Italy; ^4^ Fetal Medicine Unit, Di Venere Hospital Bari Italy; ^5^ Clinical Genetics Unit, ASST‐Spedali Civili Brescia Brescia Italy; ^6^ Department of Molecular and Translational Medicine University of Brescia Brescia Italy; ^7^ Prenatal Medicine Center, Obstetrics and Gynecology Unit, Department of Medical and Surgical Sciences for Mothers, Children and Adults University of Modena and Reggio Emilia Modena Italy; ^8^ Fetal and Perinatal Medicine and Surgery Unit, Bambino Gesù Children's Hospital, IRCCS Rome Italy; ^9^ Laboratory of Medical Genetics, Translational Cytogenomics Research Unit, Bambino Gesù Children's Hospital, IRCCS Rome Italy; ^10^ Department of Obstetrics Fondazione IRCCS San Gerardo dei Tintori Monza Italy; ^11^ School of Medicine and Surgery University of Milano‐Bicocca Monza Italy; ^12^ Fetal Medicine Unit, Department for Woman and Child Health Careggi University Hospital Florence Italy; ^13^ Department of Women and Child Health Women Health Area, Fondazione Policlinico Universitario ‘A. Gemelli’ IRCCS Rome Italy; ^14^ Fetal Medicine and Surgery, Istituto IRCCS Giannina Gaslini Genova Italy; ^15^ Department of Medicine and Surgery, Obstetrics and Gynecology Unit University of Parma Parma Italy; ^16^ Department of Women, Child and Public Health Catholic University of Sacred Heart, Fondazione Policlinico Gemelli IRCCS Rome Italy; ^17^ Department of Mother and Child Obstetrics Unit, Fondazione IRCCS Ca' Granda Ospedale Maggiore Policlinico Milan Italy; ^18^ Department of Clinical and Community Sciences University of Milan Milan Italy; ^19^ Research Center in Clinical and Translational Maternal Fetal and Neonatal Medicine University of Milan Milan Italy

**Keywords:** circulating extravillous trophoblast, clinical utility, clinically relevant CNV, fetal microimbalance, DNA‐based first‐trimester screening, genome‐wide screening, intentional screening, residual risk, single‐cell NIPT, single‐cell sequencing

## Abstract

**Objective:**

Pathogenic or likely pathogenic copy‐number variants (p/lpCNVs) are a significant cause of perinatal morbidity and mortality. Current prenatal screening based on cell‐free DNA (cfDNA) fails to detect the majority of microimbalances (microdeletions/microduplications), leaving a significant residual risk of undetected chromosomal abnormalities. This study evaluated the clinical performance of a novel single‐cell‐sequencing‐based non‐invasive prenatal testing (scsbNIPT) method utilizing circulating extravillous trophoblasts (cEVTs) for the detection of fetal p/lpCNVs, particularly microimbalances < 8 Mb.

**Methods:**

This was a prospective, blinded, observational multicenter cohort study of 1390 high‐risk pregnant women undergoing prenatal invasive diagnostic testing between November 2021 and December 2023. A 20‐mL maternal blood sample was collected from each subject between 11 + 0 and 22 + 6 weeks' gestation prior to invasive sampling. cEVTs were isolated and subjected to whole‐genome sequencing, using a proprietary workflow. scsbNIPT results were compared with standard invasive prenatal diagnostic results obtained by karyotyping and/or chromosomal microarray analysis.

**Results:**

scsbNIPT showed a sensitivity of 92.9% (95% CI, 76.5–99.1%) and a specificity of 98.2% (95% CI, 97.0–99.0%) for the detection of genome‐wide microimbalances measuring ≥ 300 kb to < 8 Mb. The sensitivity for p/lpCNVs ≥ 300 kb in pregnancies screened at 11 + 0 to 14 + 6 weeks was 100% (95% CI, 83.9–100%). For trisomy 21, the sensitivity of scsbNIPT was 98.0% (95% CI, 92.9–99.8%) and the specificity was 99.7% (95% CI, 99.0–99.9%).

**Conclusions:**

This study demonstrates the scientific validity and clinical utility of scsbNIPT for the non‐invasive detection of genome‐wide fetal p/lpCNVs, particularly microimbalances, with high sensitivity and a resolution comparable to that of chromosomal microarray analysis. scsbNIPT may offer more complete screening for genome‐wide p/lpCNVs, markedly lowering the residual risk early in pregnancy compared with existing cfDNA‐based methods. © 2026 Menarini Silicon Biosystems. *Ultrasound in Obstetrics & Gynecology* published by John Wiley & Sons Ltd on behalf of International Society of Ultrasound in Obstetrics and Gynecology.

## INTRODUCTION

Genomic microimbalances, such as microdeletions and microduplications, detected classically by chromosomal microarray analysis (CMA), are a significant cause of perinatal morbidity and mortality. Their combined prevalence substantially exceeds that of the common trisomies and is estimated to be ~1% in the general population and ~ 6% in specific high‐risk populations of pregnant women^1^, ^2^, ^3^. Genomic microimbalances are independent of maternal age, and current prenatal screening modalities often fail to detect them until late in pregnancy or postnatally^1^, ^4^, ^5^, ^6^.

Prenatal screening for fetal chromosomal abnormalities has focused historically on common autosomal trisomies. Cell‐free DNA (cfDNA)‐based non‐invasive prenatal testing (cfNIPT) has expanded to include sex‐chromosome aneuploidies and, more recently, certain large segmental imbalances greater than 7–10 Mb in size or a preselected panel of well‐known copy‐number losses^7^. However, current cfDNA‐based technologies cannot comprehensively detect microimbalances owing to limitations such as maternal DNA admixture and low sequencing depth/resolution^8^, ^9^, ^10^, ^11^, leaving a clinically relevant residual risk after a negative cfNIPT result^4^, ^12^. Cell‐based non‐invasive screening offers the prospect of analyzing 100% trophoblastic DNA, potentially enabling deeper (sequencing) analysis and earlier detection of most fetal pathogenic microimbalances^13^.

This study was conducted to compare the fetal diagnostic results from single‐cell analysis of circulating extravillous trophoblasts (cEVTs) from maternal blood with the corresponding results from chorionic villus sampling (CVS) or amniocentesis. The aim was to determine if cEVTs represent a reliable source of DNA for the non‐invasive screening of genome‐wide fetal pathogenic/likely pathogenic copy‐number variants (p/lpCNVs), particularly microimbalances < 8 Mb in size. Therefore, we assessed the clinical genomic performance of a novel workflow for the isolation and whole‐genome sequencing of individual cEVTs from maternal blood. This single‐cell‐sequencing‐based non‐invasive prenatal testing (scsbNIPT) workflow was utilized to detect p/lpCNVs (i.e. large segmental imbalances and microimbalances), common aneuploidies and fetal sex.

## METHODS

### Study design

This was a prospective observational multicenter cohort study of high‐risk pregnant women undergoing invasive diagnostic sampling at 11 prenatal diagnosis centers in Italy between November 2021 and December 2023. The initial study design was conceptualized to investigate the diagnostic performance of scsbNIPT for large chromosomal imbalances (≥ 8 Mb) and common aneuploidies as primary outcomes, and microimbalances (from < 8 Mb down to < 1 Mb) as a secondary outcome. The study protocol was subsequently amended in April 2022 (Appendix S2) to enrich the cohort with cases undergoing prenatal CMA, providing a robust comparator for the evaluation of performance with respect to microimbalances. Concurrently, analytical improvements to the proprietary copy‐number variant (CNV) calling algorithm significantly refined the detection resolution, even below 1 Mb. Therefore, while the primary and secondary study endpoints remained technically unchanged after the amendment, we shifted the primary analytical focus for this study to emphasize the clinical performance of scsbNIPT for submicroscopic p/lpCNVs, specifically microimbalances (< 8 Mb down to the maximum resolution achieved). This prioritization was based on the fact that the high resolution achieved directly addresses the unmet clinical need of early first‐trimester genome‐wide screening, which remains unattainable by current cfDNA‐based screening. The study protocol was approved by the institutional review board at each site and all participants provided written informed consent. The study was registered with ClinicalTrials.gov (NCT05671744).

Blood samples were collected by staff members at each site. Laboratory personnel processed, analyzed and interpreted single‐cell sequencing data, blinded to all clinical data except gestational age and pregnancy type (singleton or twin). The clinical staff remained blinded to the scsbNIPT results. Statistical analysis was conducted by both an independent statistician and sponsor personnel.

### Study population

Eligible participants were women aged ≥ 18 years at the time of consent with a singleton or twin pregnancy between 11 + 0 and 22 + 6 weeks' gestation, who underwent CVS or amniocentesis. Women were ineligible if they could not provide informed consent, had clinical chorioamnionitis, used illicit drugs, had known exposure to a teratogenic agent or had a known infection with a risk of vertical transmission. Gestational age was determined by crown–rump length measurement at the first‐trimester scan^14^ or by the date of embryo transfer. Sample‐size calculation was based on a 7% prevalence of cytogenetic abnormalities at the coordinating center in Milan, Italy, and a target enrolment of 1500 subjects was established (Appendix S2).

### Study procedures

From each participant, a 20 mL sample of maternal peripheral blood was collected into two CellSave™ CE‐marked tubes (Menarini Silicon Biosystems, Castel Maggiore, Italy), which were labeled with a unique identifier and sent to the research laboratory. Participants with insufficient cEVTs in the initial sample were offered a voluntary redraw.

Laboratory methods for scsbNIPT have been described previously^13^. Briefly, maternal blood samples were enriched for cEVTs using proprietary ferrofluid‐conjugated antibodies. Putative cEVTs were recovered as single cells and processed individually for whole‐genome amplification, library preparation and low‐pass whole‐genome sequencing. A proprietary computational algorithm was used to analyze the resulting next‐generation sequencing data, confirming trophoblast origin and detecting CNVs at the single‐cell level (Appendix S2). Consolidated per‐fetus data were integrated with analytical and clinical features to identify and prioritize relevant CNVs and aneuploidies (Figure S1). The scsbNIPT result was considered as screen positive if any imbalance, even in mosaic form, met quality and pathogenicity requirements (Appendix S2). The distribution of CNVs was classified based on usable cEVTs (i.e. confirmed cEVTs with a usable sequencing profile): an alteration was defined as homogeneous if detected in all individual usable cEVTs, and heterogeneous/mosaic if detected in < 100% of usable cEVTs (with subclassification as ‘cell‐private’ if the CNV was limited to a single cell). This distinction was applied only to cases for which at least two usable cEVTs were available for analysis. Cytogenetic and cytogenomic results were processed by laboratories at the enrolling centers, all of which were accredited by the Italian national health service and adhered to national cytogenomic guidelines (Appendix S2).

### Outcomes

The primary aim of this study was to demonstrate the scientific and clinical validity of scsbNIPT for the detection of p/lpCNVs, particularly microimbalances < 8 Mb. Secondary outcomes in this report included the performance of scsbNIPT for the diagnosis of common aneuploidies and the determination of fetal sex, as well as the cell isolation efficiency of the scsbNIPT technique. Common aneuploidies assessed included the common autosomal trisomies, trisomies 21 (T21), 18 and 13, as well as sex‐chromosome aneuploidies.

### Statistical analysis

We measured the concordance between the results from scsbNIPT and those from invasive prenatal diagnostic testing (CMA and/or karyotyping) by calculating sensitivity, specificity, positive predictive value (PPV) and negative predictive value.

To address intercenter variability in the classification of CNV pathogenicity, a blinded and unbiased clinical review of every fetal CNV identified by both methods was conducted. This generated a consolidated dataset of p/lpCNVs only, facilitating consistent comparison (Appendix S2). For the purpose of the study, we aimed to enrich the dataset with p/lpCNVs across various size ranges to validate the analytical performance. Therefore, any p/lpCNV that met the reporting standards of a gold‐standard invasive diagnostic CMA test was included in our clinically relevant dataset for validation, acknowledging that it does not necessarily represent the final, highly restricted clinical framework that would be implemented for prospective population screening.

The concordance analysis was stratified by diagnostic test resolution, including all p/lpCNVs ≥ 300 kb and common aneuploidies for subjects with a CMA result, but limited to large p/lpCNVs ≥ 8 Mb and common aneuploidies for subjects with only a karyotype result. Any case lacking the comparator result of the appropriate resolution (e.g. scsbNIPT showed a microimbalance < 8 Mb, but only karyotype data were available) was excluded from the analysis.

Concordance between scsbNIPT and invasive diagnostic testing was evaluated at the alteration, fetal and subject (pregnancy) levels. For the evaluation of clinical performance, the subject‐level classification was prioritized using a hierarchical adjudication algorithm. This approach accounts for the potential presence of multiple, simultaneous genomic imbalances that may vary in their concordance with the fetal genetic ‘truth’, and determines directly the overall screen‐positive or screen‐negative designation for a pregnancy. The detailed adjudication process and hierarchical algorithm are described in Appendix S2. Briefly, a true positive result was defined as detection of the identical genetic alteration by both assays, regardless of whether the pattern of alteration was homogeneous or heterogeneous/mosaic. A false positive result was defined as detection of an alteration only by scsbNIPT, and a false negative result as detection only by invasive prenatal testing. For assigning a final outcome at the subject (pregnancy) level in the presence of multiple coexisting alterations, a hierarchical priority algorithm (true positive > false negative > false positive > true negative) was utilized. Fetal sex concordance was assessed based on the presence or absence of the Y chromosome.

To evaluate cell‐isolation efficiency, we used two metrics: ‘recovery rate’, defined as the proportion of women with at least one putative cEVT recovered at cell sorting; and ‘reportable rate’, defined as the proportion of women with at least one confirmed cEVT with a usable sequencing profile. These metrics, along with the absolute count of putative and usable cEVTs, were analyzed in relation to gestational age and pregnancy type (singleton/twin) (Appendix S2).

Continuous variables are described as mean ± SD or median (interquartile range), while categorical variables are presented as *n* (%). For categorical variables, point estimates and corresponding 95% CIs were calculated using the Clopper–Pearson exact method. Group comparisons for continuous and categorical variables were performed using the Mann–Whitney *U*‐test or Kruskal–Wallis test (with Dunn's *post‐hoc* test) and Fisher's exact test, respectively. For trend analyses across ordered groups, the Jonckheere–Terpstra test was used for continuous data and the Cochran–Armitage test for proportional data. A Bonferroni correction was applied to adjust for multiple comparisons. Multivariable logistic regression analysis was employed to model the relationship between independent variables of known clinical relevance and the chance of obtaining at least one usable cEVT. Statistical significance was defined as *P* < 0.05.

## RESULTS

### Study participants and baseline characteristics

**Figure 1:**
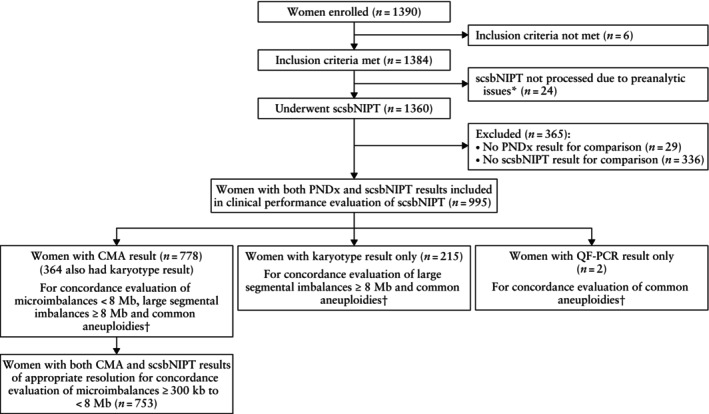
Flowchart summarizing inclusion of high‐risk pregnant women in study population. *Insufficient sample volume and/or sudden laboratory closure during COVID‐19 pandemic. †Common autosomal trisomies and sex‐chromosome aneuploidies. CMA, chromosomal microarray analysis; PNDx, prenatal diagnosis; QF‐PCR, quantitative fluorescence polymerase chain reaction; scsbNIPT, single‐cell‐sequencing‐based non‐invasive prenatal testing.

A total of 1390 women were enrolled in the study (Figure 1). Figure S2 shows a detailed breakdown of sample handling, redraw procedures and cEVT isolation (336 cases had no usable cEVTs). The primary analysis cohort comprised 995 cases, including 54 redraws (Appendix S3, Table S1, Figures S3–S5). Of these, 778 (78.2%) cases had a CMA result (CMA only or CMA plus karyotype), so they could be analyzed for all types of alteration (microimbalances, large segmental imbalances and common aneuploidies). Specifically, 753/778 (96.8%) cases were usable for the performance evaluation of scsbNIPT down to a resolution between ≥ 300 kb and < 8 Mb. The 215 cases with only a karyotype result were assessed solely for large segmental imbalances ≥ 8 Mb and common aneuploidies, while two cases with a result only from quantitative fluorescence polymerase chain reaction were restricted to the evaluation of common aneuploidies. Table 1 summarizes the baseline characteristics of the primary analysis cohort. Most participants were Caucasian (93.9%), and most pregnancies were singleton (93.1%). The detection of a fetal anomaly on ultrasound was the most common primary referral indication for invasive prenatal diagnostic testing.

**Table 1:** Demographic and clinical characteristics of 995 pregnant women included in primary analysis

Characteristic	Value
Maternal age (years)	35.11 ± 4.75
GA at blood sampling (weeks)	14.95 ± 3.14
Maternal weight (kg)	64.14 ± 12.34
Maternal height (cm)	165.10 ± 6.55
Maternal BMI (kg/m^2^)	23.54 ± 4.38
Self‐reported ethnicity/ancestry	
Caucasian	934 (93.87)
Asian	32 (3.22)
Other	29 (2.91)
Pregnancy type	
Singleton	926 (93.07)
Twin	69 (6.93)
Chorionicity in twin pregnancy	
Dichorionic	58/69 (84.06)
Monochorionic	11/69 (15.94)
Zygosity in twin pregnancy	
Dizygotic	44/69 (63.77)
Monozygotic	6/69 (8.70)
Not available	19/69 (27.54)
Mode of conception	
Spontaneous	909 (91.36)
*In‐vitro* fertilization	86 (8.64)
Principal referral indication for invasive sampling	
Fetal anomaly on ultrasound	517 (51.96)
High‐risk result at first‐trimester combined testing	232 (23.32)
Family history of genetic abnormality	146 (14.67)
Advanced maternal age (≥ 35 years)	21 (2.11)
Positive cell‐free DNA test	73 (7.34)
Personal decision (< 35 years)	6 (0.60)
Invasive sampling*	
Chorionic villous sampling	571 (57.39)
Amniocentesis	442 (44.42)
Genetic testing	
Karyotype only	215 (21.61)
CMA only	414 (41.61)
Karyotype and CMA	364 (36.58)
QF‐PCR for common aneuploidies only	2 (0.20)
Time from blood sampling to sample processing (h)	35.26 ± 18.01
Blood volume processed (mL)	19.10 ± 1.40

Data are given as mean ± SD, *n* (%) or *n*/*N* (%).

* Eighteen women underwent both methods of invasive sampling. BMI, body mass index; CMA, chromosomal microarray analysis; GA, gestational age; QF‐PCR, quantitative fluorescence polymerase chain reaction.

### Performance of scsbNIPT

**Figure 2:**
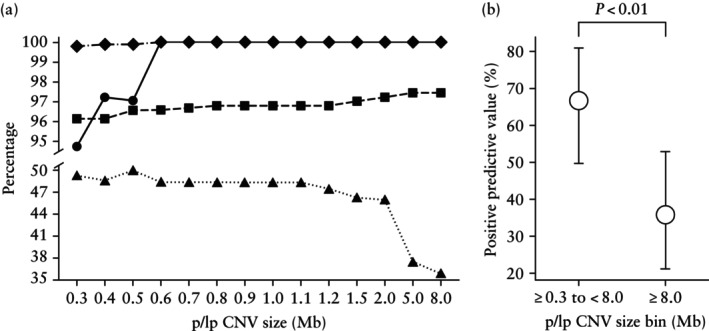
Clinical performance of single‐cell‐sequencing‐based non‐invasive prenatal testing for pathogenic and likely pathogenic copy‐number variants (p/lpCNV) according to size cut‐off in primary analysis cohort (11 + 0 to 22 + 6 weeks' gestation). (a) Sensitivity (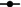), specificity (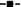), negative predictive value (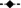) and positive predictive value (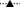) for all p/lpCNVs with a size greater than or equal to value indicated on *x*‐axis. (b) Positive predictive value with 95% CI (Clopper–Pearson) for p/lpCNVs measuring ≥ 300 kb to < 8 Mb *vs* p/lpCNVs measuring ≥ 8 Mb.

Invasive prenatal diagnostic testing detected 38/995 (3.8%) pregnancies with at least one p/lpCNV ≥ 300 kb and 137/995 (13.8%) with at least one common fetal aneuploidy. The diagnostic performance of scsbNIPT for microimbalances is reported in Table 2. The sensitivity for p/lpCNVs measuring ≥ 300 kb to < 8 Mb and those measuring ≥ 300 kb was 92.9% (95% CI, 76.5–99.1%) and 94.7% (95% CI, 82.3–99.4%), respectively. Of note, for p/lpCNVs ≥ 600 kb, the sensitivity was 100% (95% CI, 88.8–100%). The trend in clinical performance for p/lpCNVs by size cut‐off is shown in Figure 2.

**Table 2:** Clinical performance of single‐cell‐sequencing‐based non‐invasive prenatal testing for pathogenic and likely pathogenic microimbalances, large segmental imbalances and common aneuploidies in primary analysis cohort (11 + 0 to 22 + 6 weeks' gestation)

Alteration category	TP (*n*)	TN (*n*)	FP (*n*)	FN (*n*)	Sensitivity (95% CI) (%)	Specificity (95% CI) (%)	PPV (95% CI) (%)	NPV (95% CI) (%)
p/lpCNV ≥ 300 kb to < 8 Mb	26	712	13	2	92.9 (76.5–99.1)	98.2 (97.0–99.0)	66.7 (49.8–80.9)	99.7 (99.0–100)
p/lpCNV ≥ 300 kb	36	920	37	2	94.7 (82.3–99.4)	96.1 (94.7–97.3)	49.3 (37.4–61.3)	99.8 (99.2–100)
p/lpCNV ≥ 600 kb to < 8 Mb	21	723	9	0	100 (83.9–100)	98.8 (97.7–99.4)	70.0 (50.6–85.3)	100 (99.5–100)
p/lpCNV ≥ 600 kb	31	931	33	0	100 (88.8–100)	96.6 (95.2–97.6)	48.4 (35.8–61.3)	100 (99.6–100)
p/lpCNV ≥ 8 Mb	14	956	25	0	100 (76.8–100)	97.5 (96.3–98.3)	35.9 (21.2–52.8)	100 (99.6–100)
Trisomy 21	97	893	3	2	98.0 (92.9–99.8)	99.7 (99.0–99.9)	97.0 (91.5–99.4)	99.8 (99.2–100)
Trisomy 18	19	974	2	0	100 (82.4–100)	99.8 (99.3–100)	90.5 (69.6–98.8)	100 (99.6–100)
Trisomy 13	3	989	3	0	100 (29.2–100)	99.7 (99.1–99.9)	50.0 (11.8–88.2)	100 (99.6–100)
45,X	6	983	4	2	75.0 (34.9–96.8)	99.6 (99.0–99.9)	60.0 (26.2–87.8)	99.8 (99.3–100)
47,XXX	3	988	3	1	75.0 (19.4–99.4)	99.7 (99.1–99.9)	50.0 (11.8–88.2)	99.9 (99.4–100)
47,XXY	4	991	0	0	100 (39.8–100)	100 (99.6–100)	100 (39.8–100)	100 (99.6–100)
47,XYY	2	991	0	2	50.0 (6.8–93.2)	100 (99.6–100)	100 (15.8–100)	99.8 (99.3–100)
CAT + SCA	133	845	13	4	97.1 (92.7–99.2)	98.5 (97.4–99.2)	91.1 (85.3–95.2)	99.5 (98.8–99.9)
CAT + SCA + p/lpCNV ≥ 300 kb to < 8 Mb	158	805	26	6	96.3 (92.2–98.6)	96.9 (95.4–97.9)	85.9 (80.0–90.6)	99.3 (98.4–99.7)
CAT + SCA + p/lpCNV ≥ 300 kb	168	777	44	6	96.6 (92.6–98.7)	94.6 (92.9–96.1)	79.2 (73.2–84.5)	99.2 (98.3–99.7)
CAT + SCA + p/lpCNV ≥ 600 kb to < 8 Mb	153	816	22	4	97.5 (93.6–99.3)	97.4 (96.1–98.3)	87.4 (81.6–92.0)	99.5 (98.8–99.9)
CAT + SCA + p/lpCNV ≥ 600 kb	163	788	40	4	97.6 (94.0–99.3)	95.2 (93.5–96.5)	80.3 (74.1–85.5)	99.5 (98.7–99.9)

Number of pregnancies evaluated for each alteration category varies according to type of invasive prenatal testing undertaken. CAT, common autosomal trisomies; FN, false negative; FP, false positive; NPV, negative predictive value; p/lpCNV, pathogenic or likely pathogenic copy‐number variant; PPV, positive predictive value; SCA, sex‐chromosome aneuploidy; TN, true negative; TP, true positive.

Overall sensitivity and specificity of scsbNIPT for common autosomal trisomies, sex‐chromosome aneuploidies and p/lpCNVs measuring ≥ 300 kb to < 8 Mb were 96.3% (95% CI, 92.2–98.6%) and 96.9% (95% CI, 95.4–97.9%), respectively (Table 2). Sensitivity and specificity were higher in the population screened at 11 + 0 to 14 + 6 weeks, with a sensitivity of 100% (95% CI, 83.9–100%) for p/lpCNVs ≥ 300 kb (Table 3).

**Table 3:** Clinical performance of single‐cell‐sequencing‐based non‐invasive prenatal testing for pathogenic and likely pathogenic microimbalances, large segmental imbalances and common aneuploidies in first‐trimester population (screened at 11 + 0 to 14 + 6 weeks' gestation)

Alteration category	TP (*n*)	TN (*n*)	FP (*n*)	FN (*n*)	Sensitivity (95% CI) (%)	Specificity (95% CI) (%)	PPV (95% CI) (%)	NPV (95% CI) (%)
p/lpCNV ≥ 300 kb to < 8 Mb	14	363	6	0	100 (76.8–100)	98.4 (96.5–99.4)	70.0 (45.7–88.1)	100 (99.0–100)
p/lpCNV ≥ 300 kb	21	532	17	0	100 (83.9–100)	96.9 (95.1–98.2)	55.3 (38.3–71.4)	100 (99.3–100)
p/lpCNV ≥ 600 kb to < 8 Mb	9	370	4	0	100 (66.4–100)	98.9 (97.3–99.7)	69.2 (38.6–90.9)	100 (99.0–100)
p/lpCNV ≥ 600 kb	16	539	15	0	100 (79.4–100)	97.3 (95.6–98.5)	51.6 (33.1–69.8)	100 (99.3–100)
p/lpCNV ≥ 8 Mb	9	549	12	0	100 (66.4–100)	97.9 (96.3–98.9)	42.9 (21.8–66.0)	100 (99.3–100)
Trisomy 21	80	489	0	1	98.8 (93.3–100)	100 (99.2–100)	100 (95.5–100)	99.8 (98.9–100)
Trisomy 18	15	555	0	0	100 (78.2–100)	100 (99.3–100)	100 (78.2–100)	100 (99.3–100)
Trisomy 13	2	566	2	0	100 (15.8–100)	99.6 (98.7–100)	50.0 (6.8–93.2)	100 (99.4–100)
45,X	6	561	2	1	85.7 (42.1–99.6)	99.6 (98.7–100)	75.0 (34.9–96.8)	99.8 (99.0–100)
47,XXX	1	568	0	1	50.0 (1.3–98.7)	100 (99.4–100)	100 (2.5–100)	99.8 (99.0–100)
47,XXY	2	568	0	0	100 (15.8–100)	100 (99.4–100)	100 (15.8–100)	100 (99.4–100)
47,XYY	0	569	0	1	0 (0–97.5)	100 (99.4–100)	NA	99.8 (99.0–100)
CAT + SCA	105	459	4	2	98.1 (93.4–99.8)	99.1 (97.8–99.8)	96.3 (90.9–99.0)	99.6 (98.4–99.9)
CAT + SCA + p/lpCNV ≥ 300 kb to < 8 Mb	119	439	10	2	98.3 (94.2–99.8)	97.8 (95.9–98.9)	92.2 (86.2–96.2)	99.5 (98.4–99.9)
CAT + SCA + p/lpCNV ≥ 300 kb	126	425	17	2	98.4 (94.5–99.8)	96.2 (93.9–97.7)	88.1 (81.6–92.9)	99.5 (98.3–99.9)
CAT + SCA + p/lpCNV ≥ 600 kb to < 8 Mb	114	446	8	2	98.3 (93.9–99.8)	98.2 (96.6–99.2)	93.4 (87.5–97.1)	99.6 (98.4–99.9)
CAT + SCA + p/lpCNV ≥ 600 kb	121	432	15	2	98.4 (94.2–99.8)	96.6 (94.5–98.1)	89.0 (82.5–93.7)	99.5 (98.3–99.9)

Number of pregnancies evaluated for each alteration category varies according to type of invasive prenatal testing undertaken. CAT, common autosomal trisomies; FN, false negative; FP, false positive; NA, not applicable; NPV, negative predictive value; p/lpCNV, pathogenic or likely pathogenic copy‐number variant; PPV, positive predictive value; SCA, sex‐chromosome aneuploidy; TN, true negative; TP, true positive.

At the alteration level, the majority of single‐chromosome p/lpCNV false‐positive results (29/43 (67.4%)) were ≥ 8 Mb in size. These large p/lpCNVs were associated significantly with mosaic, cell‐private and terminal patterns of alteration (*P* < 0.05 for all associations) (Figure S6). Specifically, when evaluable for mosaicism, 72.0% (18/25) exhibited all three features simultaneously (mosaic, cell‐private and terminal). Reflecting these findings at the subject level, the PPV for imbalances ≥ 8 Mb was approximately half that observed for microimbalances measuring ≥ 300 kb to < 8 Mb (35.9% (95% CI, 21.2–52.8%) *vs* 66.7% (95% CI, 49.8–80.9%); *P* < 0.01) (Figure 2b). A notable proportion (5/14 (35.7%)) of unconfirmed microimbalances measuring ≥ 300 kb to < 8 Mb, counted as false positives, were detected in multiple or all cEVTs analyzed but not reported by the recruiting centers. A list of p/lpCNVs in screen‐positive cases (true and false positives) and false‐negative cases is provided in Table S2. Fetus‐ and alteration‐level results are summarized in Tables S3 and S4, respectively.

The sensitivity of scsbNIPT for T21 was 98.0% (95% CI, 92.9–99.8%), with two false‐negative results (Table 2). Fetal sex was concordant in 100% (95% CI, 99.5–100%) of cases with an available sex outcome (*n* = 796 subjects) (Table S5). The performance for sex‐chromosome aneuploidies and rare autosomal aneuploidies is reported in Tables 2 and S6, respectively.

### False‐negative cases of trisomy 21

In both cases with a false‐negative result for T21, a single confirmed cEVT was obtained. The first case involved a 33‐year‐old woman with a singleton pregnancy who underwent CVS at 12 weeks' gestation owing to increased T21 risk based on first‐trimester combined testing and a positive cfNIPT result. Cytogenetic analysis revealed T21 in all analyzed cells from both placental layers: cytotrophoblast (direct preparation) and mesenchyme (long‐term culture). In this case, the false‐negative scsbNIPT result for T21 may have been due to a cytogenetically undetected mosaic normal cell line within the cytotrophoblast layer.

The second case involved a 29‐year‐old woman with a monochorionic monoamniotic twin pregnancy. Amniocentesis was performed owing to fetal abnormalities detected on second‐trimester ultrasound and the lack of an available placental sample. Karyotype analysis detected homogeneous T21. The false‐negative scsbNIPT result for T21 was due to a mosaic placenta, either with a fully normal cytotrophoblast layer (true fetal mosaicism Type V) or a mosaic cytotrophoblast layer (true fetal mosaicism Types IV and VI).

### Extravillous trophoblast cell isolation

Recovery and reportable rates for cEVTs, together with the absolute number of usable cells, decreased with advancing gestational age (*P* < 0.0001 for all) (Figure S7). For the population screened at 11 + 0 to 14 + 6 weeks (*n* = 696), the reportable rate was 84.2%, with a mean ± SD of 4.8 ± 5.5 cells per sample (Table S7). Twin pregnancies exhibited a significantly higher number of usable cEVTs compared with singleton pregnancies (*P* < 0.0001) (Figure S8). On multivariable logistic regression analysis, twin pregnancy and gestational age at sample collection were identified as independent predictors of obtaining usable cells (Figure S9).

## DISCUSSION

This large‐scale multicenter study demonstrated that single‐cell sequencing of cEVTs isolated from maternal blood has a high sensitivity and specificity for predicting genome‐wide fetal pathogenic/likely pathogenic microimbalances measuring ≥ 300 kb to < 8 Mb, compared with invasive prenatal diagnosis using CMA. The resolution and sensitivity observed herein are currently unattainable by cfNIPT. Genome‐wide cfNIPT can detect large CNVs, but has known limitations in detecting microimbalances < 8 Mb^9^, ^10^, ^11^, ^12^, ^15^, ^16^. Since microimbalances represent the majority of pathogenic CNVs (> 80%)^5^, the residual risk associated with screen‐negative cfDNA‐based testing remains substantial. The ability of scsbNIPT to reliably detect this large, previously undetected group of p/lpCNVs shows the potential for a major reduction in the residual risk for fetal pathogenic genomic abnormalities.

With current screening modalities, less than half of fetal p/lpCNVs are detected before birth, with little or none detectable in the early first trimester^5^. Our results show that cell‐based screening would enable detection as early as the first trimester, with a cumulative sensitivity for microimbalances (≥ 300 kb to < 8 Mb) and common aneuploidies of 98.3% (95% CI, 94.2–99.8%) in the 11–14‐week screening window.

Our single‐cell study reveals important biological insights into single‐chromosome CNVs. Large segmental imbalances (≥ 8 Mb) are more likely to be false positives (mosaic, terminal, postzygotic alterations). In contrast, microimbalances (< 8 Mb) are more likely to be true positives (homogeneous, interstitial, meiotic and probably mediated by molecular mechanisms^17^). Therefore, CNV size is a relevant indicator in predicting fetal abnormality, raising questions about the utility of cfDNA screening for large segmental imbalances. While current genome‐wide cfDNA methods can screen for large p/lpCNVs (greater than 7–10 Mb in size), we emphasize that these assays share the same critical vulnerability to confined placental mosaicism (CPM)^18^, ^19^. Our single‐cell analysis provides direct biological evidence for the counterintuitive finding that larger CNVs have a lower PPV, confirming that size is linked inherently to CPM‐driven PPV concern for all non‐invasive genomic screening technologies based on analysis of the placental trophoblast layer. Importantly, unlike bulk‐analysis approaches, including both cfNIPT and bulk‐cell‐based NIPT, the single‐cell resolution of scsbNIPT provides a distinct advantage by allowing assessment of the anomaly's distribution across individual cells. This capability enables the identification of cell‐private events, which our data indicate are predominantly false positives, thereby offering a biological layer of quality control that bulk sequencing cannot provide. However, further data are required to determine how this information should be utilized optimally in the clinical management of cases presenting with these specific single‐cell findings. Our findings also suggest that CVS followed by CMA is a valid first‐line diagnostic option for screen‐positive results for microimbalances, as fetoplacental mosaicism is unlikely.

Given that our study enrolled high‐risk pregnancies, we acknowledge that a lower prevalence of genome‐wide microimbalances would be expected in the general population. However, we anticipate that the low false‐positive rate for scsbNIPT demonstrated herein should mitigate against the typical decline in PPV associated with declining prevalence, ensuring a favorable PPV even in the general prenatal population. We modeled the PPV of scsbNIPT for microimbalances (≥ 300 kb to < 8 Mb) in our high‐risk population (3.7% prevalence) for both the overall study cohort (PPV, 66.7%) and the subcohort screened at 11 + 0 to 14 + 6 weeks (PPV, 70.0%). Assuming a prevalence of microimbalances in the general prenatal population of 2.5%^1^, the corresponding modeled PPVs would be 57.0% and 61.6%, respectively. Even using a lower 1.7% prevalence associated with purely low‐risk indications^1^, the PPV would remain robust at 47.2% and 51.9%, respectively. This modeling demonstrates that the high specificity of scsbNIPT maintains a clinically favorable PPV even in low‐prevalence settings. A low false‐positive rate is attributable to minimal fetoplacental mosaicism, analysis of pure trophoblastic DNA (confirmed at the single‐cell level, which avoids problematic incidental maternal findings) and the detection of hidden dizygotic vanishing twins through a proprietary algorithm (data not shown)^20^, ^21^, ^22^. Furthermore, the false‐positive rate in this study was probably increased by microimbalances that were seen consistently in multiple (or all) cEVTs but not reported by clinical centers because of variability in CNV reporting criteria.

The clinical performance of scsbNIPT for common aneuploidies and fetal sex was generally comparable to that of cfNIPT^23^, ^24^, ^25^. We had two false‐negative results for T21, most probably due to fetoplacental mosaicism. Using a large external CVS cytogenetic dataset, in which invasive testing was performed primarily owing to advanced maternal age or parental choice, to model the residual risk in the general pregnant population for fetal aneuploidies missed owing to fetoplacental mosaicism^26^, we estimate approximately 1 in 2000 false‐negative cases for common autosomal trisomies and 1 in 1200 for sex‐chromosome aneuploidies. While the frequency of false‐negative cases for T21 observed in the present study (2/995, approximately 1/500) is numerically higher compared with the modeled residual risk for the general population (approximately 1/2000), this difference is attributable to the necessary enrichment of our high‐risk cohort.

This is the first large‐scale prospective clinical comparison of cEVT‐based screening *vs* the gold standard for the prenatal diagnosis of microimbalances. We applied our standardized clinical framework to blindly reconcile datasets from 11 clinical centers and to prioritize only clinically relevant CNVs from scsbNIPT results. This aspect is critically important in clinical practice, as untargeted genome‐wide DNA‐based screening methods necessitate a careful prioritization of clinically relevant conditions to issue a clinically actionable report, particularly in the context of prenatal screening^27^. Our future framework will prioritize p/lpCNVs with a prenatal or pediatric onset and significant morbidity. This stringent focus is essential to ensure that results remain clinically actionable and to prevent generating undue anxiety associated with detecting conditions with adult onset, thereby adhering strictly to the ethical principles governing prenatal screening.

Coorens *et al*.^28^ demonstrated the sheer extent of placental mosaicism, which is a well‐known source of false positives in cfDNA‐based testing. Our single‐cell method, however, distinguishes CNV distribution as mosaic/heterogeneous or homogeneous. The inherently low probability of random mosaicism specifically involving pathogenic/likely pathogenic regions, coupled with strict reporting of pathogenic/likely pathogenic findings^27^, ensures a low false‐positive rate. Furthermore, single‐cell sequencing offers advantages over bulk analysis^29^ by maximizing resolution, minimizing quality confounders and enhancing detection of mosaicism.

Our large dataset allowed us to associate the biology of cEVTs with pregnancy characteristics. The rates of cEVT recovery and reportability trended downward with increasing gestational age, which aligns with previous findings^13^, ^30^. In contrast to cfDNA concentrations in maternal plasma^31^, early gestational weeks (e.g. first trimester) have higher numbers of cEVTs and provide an opportune window for cell‐based screening. In addition, the number of usable cEVTs recovered is significantly higher in twin pregnancies compared with singletons. Further detailed analysis correlating the reportable rate with more extensive maternal and pregnancy characteristics, necessary for more comprehensive identification of risk factors, will be the subject of a dedicated secondary study. Extrapolating from this dataset, combined with preliminary unpublished data of 30‐mL blood draws taken before 15 weeks' gestation from the general pregnant population, we will achieve a reportable rate of > 95% from a single blood draw. These data on technology workflow advancements will be included in a future publication.

This study has several limitations. First, the rate of samples without a reportable result (24.7%) may not reflect that in the general prospective prenatal population. Our study was conducted in a high‐risk cohort with pathological referral indications and associated comorbidities. Maternal/pregnancy characteristics can affect placental invasion, vascular remodeling and, ultimately, trophoblastic cell shedding into the maternal circulation^30^, ^32^. In addition, sample redraw was performed in only a minority (36.3%) of cases for which it was indicated according to the study protocol (Figure S2).

Second, CMA was not performed for all participants following invasive sampling. Finally, the primary comparison in our study involved two different technologies (scsbNIPT and CMA) with distinct inherent resolutions and designs, which could be a potential source of non‐concordance. This aspect is critically important in prospective clinical practice, as screen‐positive results from high‐resolution scsbNIPT require confirmation using the appropriate invasive diagnostic read‐out technology (e.g. CMA covering the involved region identified in the screen).

In conclusion, this study establishes the scientific utility and validity of scsbNIPT using cEVTs for the prenatal screening of genome‐wide microimbalances measuring ≥ 300 kb to < 8 Mb, and demonstrates the potential for its use in first‐trimester screening. The resolution and sensitivity of scsbNIPT for detecting a wide range of pathogenic imbalances are unattainable with current cfNIPT technology. scsbNIPT could serve as a first‐tier universal intentional screening strategy for microimbalances in any pregnancy. Early gestation (< 11 weeks), when cEVT circulation is expected to be higher, represents an area currently unexplored by conventional screening. scsbNIPT could enable a paradigm shift in the non‐invasive diagnosis of abnormalities currently detectable only through invasive procedures and CMA at later gestational ages or postnatally.

## Supporting information


**Appendix S1** List of collaborators.
**Appendix S2** Supplementary methods.
**Appendix S3** Supplementary results.
**Figure S1** Schematic overview of clinical performance evaluation, based on comparison between single‐cell‐sequencing‐based non‐invasive prenatal testing and diagnostic gold standard.
**Figure S2** Detailed breakdown of sample handling, redraw procedures and constitution of primary analysis cohort.
**Figure S3** Distribution of first blood samplings and obtained redraws in overall cohort (*n* = 1360), according to gestational week (GW) at collection.
**Figure S4** Percentage distribution of first blood samplings and requested redraws in primary analysis cohort (*n* = 995), according to gestational week (GW) at collection.
**Figure S5** Number and proportion of cases by gestational week (GW) for first blood sampling (left) and corresponding resampling (right) in 54 cases for which redraw was obtained and usable circulating extravillous trophoblasts were isolated.
**Figure S6** Characterization of pathogenic/likely pathogenic copy‐number variants detected by single‐cell‐sequencing‐based non‐invasive prenatal testing at alteration level, based on underlying mechanism and cytogenetic features.
**Figure S7** Recovery rate, reportable rate and absolute numbers of putative and usable circulating extravillous trophoblasts isolated per subject in overall cohort (*n* = 1360), according to gestational age at sample collection.
**Figure S8** Absolute number of usable circulating extravillous trophoblasts isolated per subject, according to pregnancy type (singleton/twin) and chorionicity and zygosity in twin pregnancies.
**Figure S9** Independent predictors of obtaining usable circulating extravillous trophoblasts.
**Table S1** Gestational age distribution of subjects included in primary analysis cohort (*n* = 995).
**Table S2** List and details of all genomic imbalances detected in screen‐positive cases (true and false positives) and screen‐negative cases (false negatives) for pathogenic/likely pathogenic copy‐number variants in 995 cases included in primary analysis and in 336 cases without result from single‐cell‐sequencing‐based non‐invasive prenatal testing.
**Table S3** Performance of single‐cell‐sequencing‐based non‐invasive prenatal testing for diagnosis of pathogenic/likely pathogenic copy‐number variants and common aneuploidies, calculated at fetus level.
**Table S4** Performance of single‐cell‐sequencing‐based non‐invasive prenatal testing for diagnosis of pathogenic/likely pathogenic copy‐number variants and common aneuploidies, calculated at alteration level.
**Table S5** Performance of single‐cell‐sequencing‐based non‐invasive prenatal testing for determination of fetal sex.
**Table S6** Cumulative performance of single‐cell‐sequencing‐based non‐invasive prenatal testing for rare autosomal aneuploidies.
**Table S7** Recovery rate, reportable rate and absolute numbers of putative and usable circulating extravillous trophoblasts isolated per subject in overall cohort (*n* = 1360), according to gestational age at sample collection.

## Data Availability

De‐identified summary data (e.g., aggregated results, meta‐data) underlying the findings reported in this article are available upon reasonable request to the corresponding author (Dr. Francesca Romana Grati). Individual participant data (including sensitive data, genomic data and raw sequencing files) are not available for sharing due to the following restrictions: Ethical/Legal Constraints: Patient consent was not obtained for the deposition of individual‐level data to third‐party repositories or for sharing outside of the research team. Technical Utility: The raw data are intrinsically linked to, and functionally useless without, the proprietary analysis algorithm developed by our team. Disclosure of the raw data without the proprietary pipeline would not enable external researchers to replicate the findings or derive meaningful results.

## References

[1] Wapner RJ , Martin CL , Levy B , et al. Chromosomal microarray versus karyotyping for prenatal diagnosis. N Engl J Med. 2012;367(23):2175‐2184. 23215555 10.1056/NEJMoa1203382 PMC3549418

[2] Srebniak MI , Joosten M , Knapen MFCM , et al. Frequency of submicroscopic chromosomal aberrations in pregnancies without increased risk for structural chromosomal aberrations: systematic review and meta‐analysis. Ultrasound Obstet Gynecol. 2018;51(4):445‐452. 28556491 10.1002/uog.17533

[3] de Wit MC , Srebniak MI , Govaerts LC , et al. Additional value of prenatal genomic array testing in fetuses with isolated structural ultrasound abnormalities and a normal karyotype: a systematic review of the literature. Ultrasound Obstet Gynecol. 2014;43(2):139‐146. 23897843 10.1002/uog.12575

[4] Maya I , Salzer Sheelo L , Brabbing‐Goldstein D , et al. Residual risk for clinically significant copy number variants in low‐risk pregnancies, following exclusion of noninvasive prenatal screening–detectable findings. Am J Obstet Gynecol. 2022;226(4):562.e1‐562.e8. 10.1016/j.ajog.2021.11.016 34762861

[5] Gadsbøll K , Vogel I , Kristensen SE , et al. Combined first‐trimester screening and invasive diagnostics for atypical chromosomal aberrations: Danish nationwide data on prenatal profiles and detection compared with NIPT. Ultrasound Obstet Gynecol. 2024;64(4):470‐479. 38642365 10.1002/uog.27667

[6] Palmer LD , Butcher NJ , Boot E , et al. Elucidating the diagnostic odyssey of 22q11.2 deletion syndrome. Am J Med Genet A. 2018;176(4):936‐944. 29575622 10.1002/ajmg.a.38645 PMC5873609

[7] Fiorentino D , Dar P‘e . Prenatal screening for microdeletions and rare autosomal aneuploidies. Clin Obstet Gynecol. 2023;66(3):579‐594. 37438896 10.1097/GRF.0000000000000799

[8] Grati FR , Gross SJ . Noninvasive screening by cell‐free DNA for 22q11.2 deletion: benefits, limitations, and challenges. Prenat Diagn. 2019;39(2):70‐80. 30625249 10.1002/pd.5391

[9] Lo KK , Karampetsou E , Boustred C , et al. Limited clinical utility of non‐invasive prenatal testing for subchromosomal abnormalities. Am J Hum Genet. 2016;98(1):34‐44. 26708752 10.1016/j.ajhg.2015.11.016 PMC4716686

[10] Zhao C , Tynan J , Ehrich M , et al. Detection of fetal subchromosomal abnormalities by sequencing circulating cell‐free DNA from maternal plasma. Clin Chem. 2015;61(4):608‐616. 25710461 10.1373/clinchem.2014.233312

[11] Kucharik M , Gnip A , Hyblova M , et al. Non‐invasive prenatal testing (NIPT) by low coverage genomic sequencing: Detection limits of screened chromosomal microdeletions. PLoS One. 2020;15(8):e0238245. 32845907 10.1371/journal.pone.0238245 PMC7449492

[12] Guseh S , Wilkins‐Haug L , Kaimal A , et al. Utility of noninvasive genome‐wide screening: a prospective cohort of obstetric patients undergoing diagnostic testing. Genet Med. 2021;23(7):1341‐1348. 33782554 10.1038/s41436-021-01147-4

[13] Doffini A , Forcato C , Mangano C , et al. Isolation of single circulating trophoblasts from maternal circulation for noninvasive fetal copy number variant profiling. Prenat Diagn. 2023;43(1):14‐27. 36443901 10.1002/pd.6275 PMC10107339

[14] Italian Society of Ultrasound in Obstetrics and Gynecology (SIEOG) . National guidelines for ultrasound in obstetrics and gynaecology. https://www.sieog.it/wp‐content/uploads/2021/11/Guidelines‐for‐obstetric_compressed.pdf 10.1016/j.ejogrb.2022.10.016 36351360

[15] Lefkowitz RB , Tynan JA , Liu T , et al. Clinical validation of a noninvasive prenatal test for genomewide detection of fetal copy number variants. Am J Obstet Gynecol. 2016;215(2):227.e1‐227.e16. 10.1016/j.ajog.2016.02.030 26899906

[16] Ye X , Lin S , Song X , et al. Identification of copy number variants by NGS‐based NIPT at low sequencing depth. Eur J Obstet Gynecol Reprod Biol. 2021;256:297‐301. 33310305 10.1016/j.ejogrb.2020.11.026

[17] Harel T , Lupski JR . Genomic disorders 20 years on‐mechanisms clinical manifestations. Clin Genet. 2018;93(3):439‐449. 28950406 10.1111/cge.13146

[18] Chen Y , Yu Q , Mao X , Lei W , He M , Lu W . Noninvasive prenatal testing for chromosome aneuploidies and subchromosomal microdeletions/microduplications in a cohort of 42,910 single pregnancies with different clinical features. Hum Genomics. 2019;13(1):60. 31783780 10.1186/s40246-019-0250-2 PMC6884830

[19] van der Meij KRM , Sistermans EA , Macville MVE , et al. National implementation of genome‐wide non‐invasive prenatal testing as a first‐tier screening test in the Netherlands. Am J Hum Genet. 2019;105(6):1091‐1101. 31708118 10.1016/j.ajhg.2019.10.005 PMC6904791

[20] Grati FR , Malvestiti F , Ferreira JCPB , et al. Fetoplacental mosaicism: potential implications for false‐positive and false‐negative noninvasive prenatal screening results. Genet Med. 2014;16(8):620‐624. 24525917 10.1038/gim.2014.3

[21] Raymond Y , Fernando S , Menezes M , et al. Placental, maternal, fetal, and technical origins of false‐positive cell‐free DNA screening results. Am J Obstet Gynecol. 2024;230(4):381‐389. 38008147 10.1016/j.ajog.2023.11.1240

[22] Turriff AE , Annunziata CM , Malayeri AA , et al. Prenatal cfDNA sequencing and incidental detection of maternal cancer. N Engl J Med. 2024;391(22):2123‐2132. 39774314 10.1056/NEJMoa2401029 PMC11711700

[23] Bussolaro S , Raymond YC , Acreman ML , et al. The accuracy of prenatal cell‐free DNA screening for sex chromosome abnormalities: a systematic review and meta‐analysis. Am J Obstet Gynecol MFM. 2023;5(3):100844. 36572107 10.1016/j.ajogmf.2022.100844

[24] Rose NC , Barrie ES , Malinowski J , et al. Systematic evidence‐based review: the application of noninvasive prenatal screening using cell‐free DNA in general‐risk pregnancies. Genet Med. 2022;24(7):1379‐1391. 35608568 10.1016/j.gim.2022.03.019

[25] Gil MM , Galeva S , Jani J , et al. Screening for trisomies by cfDNA testing of maternal blood in twin pregnancy: update of The Fetal Medicine Foundation results and meta‐analysis. Ultrasound Obstet Gynecol. 2019;53(6):734‐742. 31165549 10.1002/uog.20284

[26] Benn P , Malvestiti F , Grimi B , Maggi F , Simoni G , Grati FR . Rare autosomal trisomies: comparison of detection through cell‐free DNA analysis and direct chromosome preparation of chorionic villus samples. Ultrasound Obstet Gynecol. 2019;54(4):458‐467. 31237735 10.1002/uog.20383

[27] Murray MF , Giovanni MA , Doyle DL , et al. DNA‐based screening and population health: a points to consider statement for programs and sponsoring organizations from the American College of Medical Genetics and Genomics (ACMG). Genet Med. 2021;23(6):989‐995. 33727704 10.1038/s41436-020-01082-w

[28] Coorens THH , Oliver TRW , Sanghvi R , et al. Inherent mosaicism and extensive mutation of human placentas. Nature. 2021;592(7852):80‐85. 33692543 10.1038/s41586-021-03345-1 PMC7611644

[29] Hatt L , Ravn K , Dahl Jeppesen L , et al. How does cell‐based non‐invasive prenatal test (NIPT) perform against chorionic villus sampling and cell‐free NIPT in detecting trisomies and copy number variations? A clinical study from Denmark. Prenat Diagn. 2023;43(7):854‐864. 37199490 10.1002/pd.6387

[30] Vossaert L , Chakchouk I , Zemet R , Van den Veyver IB . Overview and recent developments in cell‐based noninvasive prenatal testing. Prenat Diagn. 2021;41(10):1202‐1214. 33974713 10.1002/pd.5957 PMC9355411

[31] Wang E , Batey A , Struble C , Musci T , Song K , Oliphant A . Gestational age and maternal weight effects on fetal cell‐free DNA in maternal plasma. Prenat Diagn. 2013;33(7):662‐666. 23553731 10.1002/pd.4119

[32] Brosens I , Pijnenborg R , Vercruysse L , Romero R . The “Great Obstetrical Syndromes” are associated with disorders of deep placentation. Am J Obstet Gynecol. 2011;204(3):193‐201. 21094932 10.1016/j.ajog.2010.08.009 PMC3369813

