# Supplementary material for: Single‐cell‐based non‐invasive screening for fetal pathogenic microimbalances using maternal blood: comparison with invasive prenatal diagnosis
Source: Ultrasound Obstet Gynecol. 2026 Mar 25;67(4):500–9. doi: 10.1002/uog.70201 (PMC13040114; doi:10.1002/uog.70201)
Supplement: Supplementary file 1 — Appendix S1 List of collaborators. Appendix S2 Supplementary methods. Appendix S3 Supplementary results. Figure S1 Schematic overview of clinical performance evaluation, based on comparison between single‐cell‐sequencing‐based non‐invasive prenatal testing and diagnostic gold standard. Figure S2 Detailed breakdown of sample handling, redraw procedures and constitution of primary analysis cohort. Figure S3 Distribution of first blood samplings and obtained redraws in overall cohort (n = 1360), according to gestational week (GW) at collection. Figure S4 Percentage distribution of first blood samplings and requested redraws in primary analysis cohort (n = 995), according to gestational week (GW) at collection. Figure S5 Number and proportion of cases by gestational week (GW) for first blood sampling (left) and corresponding resampling (right) in 54 cases for which redraw was obtained and usable circulating extravillous trophoblasts were isolated. Figure S6 Characterization of pathogenic/likely pathogenic copy‐number variants detected by single‐cell‐sequencing‐based non‐invasive prenatal testing at alteration level, based on underlying mechanism and cytogenetic features. Figure S7 Recovery rate, reportable rate and absolute numbers of putative and usable circulating extravillous trophoblasts isolated per subject in overall cohort (n = 1360), according to gestational age at sample collection. Figure S8 Absolute number of usable circulating extravillous trophoblasts isolated per subject, according to pregnancy type (singleton/twin) and chorionicity and zygosity in twin pregnancies. Figure S9 Independent predictors of obtaining usable circulating extravillous trophoblasts. Table S1 Gestational age distribution of subjects included in primary analysis cohort (n = 995). Table S2 List and details of all genomic imbalances detected in screen‐positive cases (true and false positives) and screen‐negative cases (false negatives) for pathogenic/likely pathogenic copy‐number varian [file UOG-67-500-s001.docx]

**Supplementary Material**

**Table of contents**

[1.](#_heading=h.8l7chvq5qakt) Appendix S1 List of collaborators 3

[2.](#_heading=h.5dk9n5aa1lhb) Appendix S2 Supplementary methods 4

[2.1.](#_heading=h.htbucnobq4ei) Trial registration 4

[2.2.](#_heading=h.4sy3aiqj8q6d) Evolution of the study protocol and NGS data analysis 5

[2.3.](#_heading=h.gd08pjbun9vn) Sample size calculation 6

[2.4.](#_heading=h.hl2u4qqj0pe5) Single-cell sequencing-based NIPT laboratory process 7

[2.5.](#_heading=h.v9z6n3c2vy7y) Invasive Diagnostic testing 8

[2.5.1.](#_heading=h.chbq5jsix09) Chromosomal Microarray 8

[2.5.2.](#_heading=h.mnzwtqusf250) Cytogenetic analysis 9

[2.6.](#_heading=h.3rzab9po0xi6) Blinded Unbiased Clinical Curation of the Clinical and scsbNIPT Databases of detected CNVs 9

[2.7.](#_heading=h.4x1b9dfseg9k) Hierarchical priority algorithm and concordance adjudication process 11

[2.7.1.](#_heading=h.pn0750ic1d3q) Pathogenic and Likely Pathogenic Copy Number Variations Analysis 13

[2.7.2.](#_heading=h.yjq9vj8g7wf3) Aneuploidy Analysis 13

[2.8.](#_heading=h.4isrnzkeejgc) Fetal cell isolation 14

[2.9.](#_heading=h.tf654iqc2yaa) Definition of outcomes, exposures, predictors, potential confounders, effect modifiers, diagnostic criteria and missing data 14

[3.](#_heading=h.x9ki2kc9iy2l) Appendix S3 Supplementary Results 15

[3.1.](#_heading=h.xh68bwtkjumv) Breakdown of sample handling, redraw procedures, and the constitution of the primary analysis cohort 15

[4. Figures 18](#_heading=h.67sfs0c8zsw7)

[4.1.](#_heading=h.ilcekrrkzcr0) Figure S1 18

[4.2.](#_heading=h.bz44wwsoo4dg) Figure S2 19

[4.3.](#_heading=h.hqsckeqaa605) Figure S3 20

[4.4.](#_heading=h.r10ab5hyzupq) Figure S4 21

[4.5.](#_heading=h.bcpqts5si6f9) Figure S5 22

[4.6.](#_heading=h.7np9yi2twrn0) Figure S6 23

[4.7.](#_heading=h.crqb9aq7i45v) Figure S7 25

[4.8.](#_heading=h.6oa5lf1erg6z) Figure S8 27

[4.9.](#_heading=h.o5p5ncs0d7x4) Figure S9 28

[5.](#_heading=h.8820t2b62n72) Tables 29

[5.1.](#_heading=h.u6b30w1esynv) Table S1 29

[5.2.](#_heading=h.jtrm07duafd5) Table S2 30

[5.3.](#_heading=h.ajga9ycz4ktt) Table S3 43

[5.4.](#_heading=h.uehuntxcv5kc) Table S4 44

[5.5.](#_heading=h.v4nl814ivgca) Table S5 47

[5.6.](#_heading=h.fw598fqi89k) Table S6 48

[5.7.](#_heading=h.snejvets719x) Table S7 49

[6.](#_heading=h.fnaputjqfd0j) References 50

1. **Appendix S1 List of collaborators**

*For the ‘Single-cell Based Noninvasive Screening’ trial Investigators:*

- Single-cell sequencing-based NIPT proprietary workflow development and study samples analysis
  - Reproductive Precision Medicine Unit, Menarini Silicon Biosystems, Castel Maggiore (Bologna), Italy: ***Emilia Dora Giovannone MSc, Ph.D., Chiara Mangano MSc, Ph.D., Chiara Maranta MSc, Camilla Amadesi MSc, Antonio Brocco MSc, Arianna Casadei MSc, Melissa Garrì MSc, Rebecca Maiocchi MSc, Ph.D., Ilaria Molinaro MSc, Elisa Ortolan MSc, Angela Piano MSc, Ph.D., Maria Chiara Iannitiello MSc, Davide Lisi MSc, and Agnese Feresin MD***
- Patient accrual, dataset management and prenatal genetic diagnosis:
  - Trieste: ***Paolo Gasparini MD, Ph.D.,*** Department of Medical Genetics; Institute for Maternal and Child Health-IRCCS, Burlo Garofolo, Trieste, Italy; Department of Medicine, Surgery and Health Sciences, University of Trieste, Trieste, Italy; ***Camilla Fregona MD,*** Unit of Fetal Medicine and Prenatal Diagnosis, Institute for Maternal and Child Health IRCCS Burlo Garofolo, Trieste, Italy; Department of Medicine, Surgery and Health Sciences, University of Trieste, Trieste, Italy.
  - Bari: ***Georgios Rembouskos MD****,* Fetal Medicine Unit, Di Venere Hospital, Bari, Italy.
  - Brescia: ***Franco Edoardo Odicino MD****,* Department of Clinical and Experimental Sciences, University of Brescia, Brescia, Italy; ***Marino Signorelli MD****,* Prenatal Diagnosis Unit, Department of Obstetrics and Gynecology, ASST-Spedali Civili of Brescia, Italy.
  - Modena: ***Alessandra Sponzilli MD*** and ***Fabio Facchinetti MD,*** Obstetrics and Gynecology Unit, Departments of Medical and Surgical Sciences for Mothers, Children and Adults, University of Modena and Reggio Emilia, Modena, Italy
  - Roma OPBG and San Pietro: ***Chiara Vassallo MD****,* Fetal and Perinatal Medicine and Surgery Unit, Area of Fetal, Neonatal and Cardiological Sciences, Bambino Gesù Children's Hospital and Research Institute, Rome, Italy; ***Elena Nicastri MD****,* Fetal and Perinatal Medicine and Surgery Unit, Area of Fetal, Neonatal and Cardiological Sciences, Bambino Gesù Children's Hospital and Research Institute, Rome, Italy; Obstetrics and Gynecology Unit, Maternal and Child Department, San Pietro Fatebenefratelli Hospital, Rome, Italy; ***Maria Grazia Di Gregorio MD,*** Medical Genetic Unit San Pietro Fatebenefratelli Hospital, Rome, Italy; ***Valeria Orlando MSc,*** Laboratory of Medical Genetics, Translational Cytogenomics Research Unit, Bambino Gesù Children's Hospital, IRCCS, Rome, Italy.
  - Monza: ***Sabrina Cozzolino MD, PhD*** and ***Maria Verderio, MD PhD*** Department of Obstetrics, Foundation IRCCS San Gerardo dei Tintori, Monza, Italy;
  - Firenze: ***Giulia Masini MD*** and ***Chiara Franchi MD***, Fetal Medicine Unit, Department for Woman and Child Health, Careggi University Hospital, Florence, Italy.
  - Roma Gemelli: ***Francesco Danilo Tiziano MD***, Complex Unit of Medical Genetics, Fondazione Policlinico Agostino Gemelli IRCCS, Rome, Italy; Section of genomic medicine, Department of life sciences and public health, Catholic University, Rome, Italy; ***Domizia Pasquetti MD***, Complex Unit of Medical Genetics, Fondazione Policlinico Agostino Gemelli IRCCS, Rome, Italy.
  - Genova: ***Giulia Biancotto MD***, Dept. Obstetrics and Gynecology, Azienda Ospedaliera Universitaria Integrata Verona, Verona University, Verona, Italy; ***Francesca Della Sala MD***, Dept. Obstetrics and Gynecology, San Paolo Hospital, Polo Universitario ASST Santi Paolo e Carlo, Milano, Italy.
  - Parma: ***Nicola Volpe MD, Ph.D.,*** Fetal Medicine Center, Obstetrics and Gynecology Unit, University Hospital of Parma, Parma, Italy; ***Andrea Dall'Asta MD, Ph.D.,*** Department of Medicine and Surgery, Obstetrics and Gynecology Unit, University of Parma; Fetal Medicine Center, Obstetrics and Gynecology Unit, University Hospital of Parma, Parma, Italy.
  - Milano: ***Rosamaria Silipigni MSc, CLG*** and ***Ilaria Catusi, MSc, CLG, Ph.D.*** Laboratorio di Genetica Medica della Fondazione IRCCS Ca' Granda Ospedale Maggiore Policlinico

1. **Appendix S2 Supplementary methods**
   1. Trial registration

The design of this study was strictly that of an observational, prospective cohort study aimed at evaluating the analytical performance and concordance of the scsbNIPT test against gold-standard invasive diagnostic results. Crucially, the clinical management of any pregnancy in this cohort was never altered based on scsbNIPT results, nor were test results communicated to clinicians or pregnant women during the study period.

However, the coordinating center's Ethics Committee classified the study as an interventional clinical trial from a strictly procedural and liability standpoint. This classification was mandated due to the collection of an additional 20 mL blood sample—a procedure outside of the patients' routine standard of care—and was considered necessary for final ethics approval. Consequently, the study was registered on ClinicalTrials.gov as an interventional trial to maintain consistency with the approved protocol document. Therefore this dichotomy between the study’s clinical design (observational) and its administrative registration (interventional) is an administrative necessity in certain clinical research settings.

- 1. Evolution of the study protocol and NGS data analysis

Originally, the primary objective was to assess single-cell sequencing-based noninvasive prenatal testing (scsbNIPT) performance for all fetal chromosomal imbalances >8Mb, including trisomy 21 (T21) and other common aneuploidies, in singletons and twins. Secondary objectives were to assess 1-8Mb pathogenic/likely pathogenic copy number variations (P/LP CNVs), fetal sex, and the technology's effectiveness for fetal cell isolation. The study protocol did not specify the type of fetal invasive diagnostic procedure assay (e.g., karyotype or chromosomal microarray analysis, CMA). Therefore the protocol was amended in April 2022 to: (a) enrich the population for CMA inclusion for increased comparison of submicroscopic imbalances; (b) modify blood redraw criteria based on the number of putative fetal cells initially recovered (from 0 to less than two in singletons or three in twins) and (c) extend the gestational age window for blood redraw from 21 to 22+6 weeks.

Therefore, although the primary and secondary endpoints remained unchanged, with protocol amendment the study's focus expanded to include a more robust investigation of micro-imbalances (<8Mb) with clear or highly likely clinical significance. This expansion was enabled by increasing the proportion of enrolled cases with CMA as the diagnostic reference readout.

Analyzing micro-imbalances necessitated revisions to the original analytical pipeline, which was primarily designed for aneuploidy and large imbalance (>8Mb) detection. To address this, the copy number calling algorithm was refined to improve signal detection and, consequently, resolution, with the aim of enhancing sensitivity and specificity for smaller imbalances, including those below 1Mb. After completion of the initial enrollment and primary analysis using the original algorithm, all study data underwent a comprehensive reanalysis using the updated algorithm. This reanalysis was performed while maintaining blinded diagnostic outcomes and focused on P/LP CNVs in addition to large imbalances and aneuploidies. However, heterogeneity in pathogenicity classification among enrolling sites, particularly in high-risk indications, led to substantial variability in the reporting of small alterations, including variants of uncertain significance (VOUS). Consequently, to ensure a standardized comparison between CMA diagnostic results and those from the scsbNIPT algorithm, a manual review of all alterations detected by CMA was deemed necessary and incorporated into the reanalysis. This review applied the same clinical criteria used for classifying scsbNIPT alterations and was performed while maintaining blinding to the comparator results ensuring an unbiased comparison between CMA and scsbNIPT results limited to P/LP CNVs.

- 1. Sample size calculation

In the original protocol, the prevalence of cytogenetically visible chromosome abnormalities on chorionic villi and amniotic fluid samples at the coordinating center was conservatively adjusted to 7% for sample size calculations, assuming a sensitivity of 90%, an accuracy of ±7%, and a 95% confidence level. Under these assumptions, the required sample size was calculated to be 1,008 women. This sample size was deemed sufficient to ensure that, with the same level of precision, the study would be adequately powered to conservatively assess specificity at any level greater than 90%. Preliminary data showed that the recovery of at least one cEVT would be achievable in 75% of the collected samples^1^. Accordingly, to ensure adequate statistical power, the study aimed to recruit 1,344 pregnant women (calculated as 1,008 divided by 0.75). Taking into account a 10% failure rate due to potential dropouts or unreliable samples (e.g., low-quality DNA, experimental failure), the target was adjusted to a minimum of 1,500 women.

At protocol amendment, we verified the projected accuracy of sensitivity and specificity achievable with the previously calculated sample size, adjusting only for changes in the prevalence of P/LP CNVs. We used a conservative prevalence estimate of 3%, derived from the assumption that 50% of the cohort would be classified as low-risk based on prenatal screening or advanced maternal age, and 50% would have undergone invasive procedures due to fetal ultrasound abnormalities. This 3% blended prevalence estimate was informed by literature values of approximately 1% P/LP CNV prevalence in low-risk populations and 6% in cases with fetal anomalies^2,3^. This would enable the estimation of sensitivity and specificity for P/LP CNV detection with accuracies of ±10% and ±2%, respectively.

- 1. Single-cell sequencing-based NIPT laboratory process

Samples underwent scsbNIPT processing within 96h from collection. Maternal blood samples were enriched for fetal cEVTs using proprietary ferrofluid‐conjugated antibodies and a custom protocol on the automated CellTracks AutoPrep System (Menarini Silicon Biosystems). Putative cEVTs were recovered as single cells and processed individually for whole-genome amplification and library preparation (*Ampli One™* Whole Genome Amplification (WGA) and *Ampli One™* LowPass kit, Menarini Silicon Biosystems). The procedure was automated on Hamilton Liquid Handling Robots (Hamilton Bonaduz AG, Switzerland). Libraries were sequenced by an external service using low-pass whole genome sequencing with ~4M reads/sample on average (NovaSeq6000, Illumina). Raw next generation sequencing (NGS) data were processed using a proprietary in-house pipeline designed for single-cell copy number variation (CNV) detection. After aligning sequencing reads to the hg19 human reference genome, bias from the Ampli1 amplification was mitigated by normalizing the read counts within 100kb genomic bins. The normalized data was then segmented to identify potential CNVs, which were subsequently filtered for statistical significance using a two-tailed Mann-Whitney U test (p < 0.01). The same NGS data was used to confirm the fetal origin of each cell via allelic content analysis, thereby ensuring that result comparison with gold-standard prenatal diagnosis was performed on actual fetal cells. Following single-cell CNV calling, data derived from multiple cells originating from the same fetus were aggregated to enhance the reliability and confidence of CNV detection (Fig S1). Finally, all detected CNVs underwent clinical assessment to identify and prioritize for reporting those classified as LP/P in accordance with established clinical guidelines and consolidated clinical framework [see next paragraph]. This comprehensive evaluation allowed the stratification of detected CNVs into three distinct confidence levels: high, medium, and low. Notably, only those ones categorized as having medium or high confidence levels were utilized for the comparative analysis with prenatal diagnosis results.

- 1. Invasive Diagnostic testing
     1. *Chromosomal Microarray*

DNA extraction was performed according to local protocols. Chromosomal microarray assays were performed according to the manufacturer’s protocol. Array platforms used were the following ones:

- Agilent Array CGH (Sites: 380-01, 380-02, 380-03, 380-05, 380-06, 380-08, 380-11)
- Sureprint G3 Human CGH microarray Kit, 8x60K
- SurePrint G3 Human CGH Microarray Kit, 4x180K
- GenetiSure Cyto 8x60K CGH
- GenetiSure Cyto 4x180K CGH+SNP Microarray kit
- Illumina SNP array (Site: 380-10, 380-04)
- Infinium CytoSNP-850K
- Infinium Human OmniExpress Exome BeadChip
- CytoSure Array CGH (OGT) (Site: 380-10)
- CytoSure v2 array 4x180k
  - 1. *Cytogenetic analysis*

Cytogenetic testing was performed consistent with Italian guidelines and described in Grati et al, 2022^4^. For chorionic villi samples (CVS), cytotrophoblast analysis was based on direct (DIR) uncultured villus cells and/or mesenchyme based on long-term culture (LTC). When both direct (DIR) and LTC were performed, at least 16 metaphases were scored and analyzed (6 cells from DIR and 10 cells from LTC). For AF samples, 10 metaphases were analysed from 10 colonies from at least two independent cultures when in situ method was applied, or, 16 metaphases from at least two independent cultures when suspension culture method was used.

In cases of suspected mosaicism (e.g.: after detection of a mosaicism in CV or a high risk for aneuploidies at NIPT in presence of a normal standard karyotype), all laboratories applied an extended work-up on the AF sample (50 metaphases from 24 colonies from independent cultures and/or interphase FISH performed on 50-100 nuclei)^5,6^. The karyotype description followed the current International System for Human Cytogenomic Nomenclature (ISCN) recommendations^7^.

- 1. Blinded Unbiased Clinical Curation of the Clinical and scsbNIPT Databases of detected CNVs

Manual blinded curation of both clinical and scsbNIPT databases of detected CNVs was based on a standardized, homogeneous and evidence-based clinical framework for genome-wide fetal-CNV reporting that carefully prioritizes clinically relevant findings. CNVs were classified as clinically relevant (P/LP) if they: (1) overlapped or encompassed previously described disease-associated genes and critical regions of well-known contiguous gene syndromes, or (2) encompassed a defined set of dosage-sensitive disease-associated regions. The curation process integrated the joint criteria of the American College of Medical Genetics and Genomics (ACMG) and the Clinical Genome Resource (ClinGen) for the classification of constitutional CNVs in the context of neurodevelopmental disorders, multiple congenital anomalies, and ultrasound abnormalities^8^. Additionally, it involved a systematic survey, curation, and harmonization of publicly available repositories, including but not limited to the Human Phenotype Ontology, GeneReviews, Online Mendelian Inheritance in Man (OMIM), ClinGen, Database of Genomic Variants (DGV), PubMed, and Google Scholar. This comprehensive review specifically focused on the clinical effects of dosage changes (haploinsufficiency and triplosensitivity).

For the purpose of this study, we aimed to enrich the dataset with P/LP CNVs across various size ranges. Therefore, any P/LP CNV that met the reporting standards of a gold-standard CMA invasive diagnostic test, including those with adult-onset or variable penetrance, was included in our clinically relevant set for validation. This comprehensive approach was necessary to validate the analytical performance of the scsbNIPT across the full spectrum of detectable P/LP aberrations, but it does not represent the final, highly restricted clinical framework that will be implemented for prospective population screening.

The four primary functional classification categories of detected P/LP CNVs are defined as follows:

- Early Onset (EO): overlapping with a dosage-sensitive critical region or containing at least one gene robustly associated with a condition that typically manifests within the prenatal-to-pediatric age range.
- Late Onset (LO): containing one or more genes robustly associated with conditions that typically manifest in adulthood.
- Susceptibility Locus (S): CNVs that confer an increased risk or predisposition to a condition but are not, by themselves, considered strictly causative.
- Variable Penetrance (VP): associated with conditions where the likelihood of clinical manifestation is known to be incomplete and consistently reported in literature.

The classification of P/LP CNVs into these different phenotypic categories was guided by the following criteria:

- prioritising the most severe potential outcome: when a CNV contained multiple genes associated with different types of onset, the classification defaulted to the most clinically severe category (EO>LO>VP>S);
- large CNVs, such as those containing numerous protein-coding genes (≥20) were classified EO;
- diseases with onset in puberty/adolescence were conservatively classified as LO;
- CNVs that constitute a carrier state are classified as S.

Once finalized, the curated clinical diagnostic database represented the ground truth, used for the concordance adjudication by comparison with the curated scsbNIPT result database.

- 1. Hierarchical priority algorithm and concordance adjudication process

Diagnostic performance of scsbNIPT was quantified by calculating sensitivity, specificity, positive predictive value (PPV), and negative predictive value (NPV) using all usable cEVTs regardless of the type of sampling (first or resampling). These metrics were derived from the number of true positives (TP), true negatives (TN), false positives (FP), and false negatives (FN). Adjudication criteria were established as follows:

- If the same alteration was present in both assay results, even in mosaic form, it was classified as “concordant positive” or TP (e.g. prenatal diagnosis detected a mosaic Trisomy 21 and scsbNIPT detected a homogeneous Trisomy 21);
- In cases of discordance, where prenatal diagnostic results were normal but scsbNIPT indicates an abnormality, a "discordant positive" (false positive, FP) result was assigned.
- Conversely, if prenatal diagnostic results were abnormal while scsbNIPT was normal, a "discordant negative" (false negative, FN) result was assigned;
- If no alterations were detected in both scsbNIPT and invasive diagnostic testing, a "concordant negative" (true negative, TN) adjudication was assigned.

To evaluate concordance between scsbNIPT and diagnostic testing, a multi-level approach was adopted, assessing data at alteration, fetal, and subject (pregnancy) levels. Subject and fetal level assessment was complicated by the co-existence of multiple genomic imbalances within a single subject (pregnancy) or fetus. These imbalances could vary in their concordance with diagnostic results; for instance, a single fetus/pregnancy might simultaneously present with:

- A false negative (FN): an imbalance detected only by diagnostic testing.
- A false positive (FP): an imbalance detected only by scsbNIPT.
- A true positive (TP): an imbalance detected by both methods.

To derive a single, definitive subject-level (or fetal-level) classification essential for clinical utility and performance assessment, a standardized hierarchical priority algorithm was implemented.

The hierarchical priority algorithm for assigning a unique outcome in cases involving multiple alterations was as follows:

- TP priority: If any alteration was identified by both methods — regardless of other alterations present — the scsbNIPT was classified as TP.
- FN priority: In the absence of a TP alteration, if any FN alteration was identified, the scsbNIPT result was classified as FN.
- FP priority: In the absence of TP and FN alterations, if any FP alteration was identified, the scsbNIPT result was classified as FP.
- TN priority: If no alterations were identified by either scsbNIPT or diagnostic testing, the scsbNIPT result was classified as true negative (TN).

Consequently, because of this subject-level concordance evaluation, the total number of individual abnormalities detected across the overall cohort differs from the number of subjects classified as abnormal, as one pregnancy may harbor multiple imbalances.

To account for design and resolution differences between scsbNIPT and invasive diagnostic testing, several factors were considered in the performance calculations. First, given variations in assay design, a 30% reciprocal overlap of genomic coordinates was deemed sufficient for classifying P/LP CNVs (‘large’ or ’micro’) as true positives (TP). Second, to perform a robust tier-based analysis, discrepancies in resolution between the two methods were addressed by limiting concordance assessment to imbalances within the size range detectable by both. Specifically, regarding scsbNIPT, a subject-specific resolution criterion was applied to define eligible subjects and assessable alterations. Similarly, when limited by the resolution of “ground truth” methods (e.g., if only karyotyping was available), the assessment focused solely on compatible targets, such as aneuploidies and large imbalances (>8Mb). As a direct consequence of this resolution-based filtering, the sample size varies across analytical tiers (n=995 for CNVs ≥ 300kb or aneuploidies; n=753 for CNV 300kb–8Mb). ScsbNIPT technology performance was evaluated for LP/P CNVs and for aneuploidies separately and in aggregate, providing a measure of the scsbNIPT test performance at the subject level both in all gestational age (11-22) and in the first trimester only (11-14 weeks).

- - 1. *Pathogenic and Likely Pathogenic Copy Number Variations Analysis*

The CNV panel included the following size-defined categories:

- P/LP CNVs between 300Kb and 8Mb.
- P/LP CNVs ≥300Kb
- P/LP CNVs between 600Kb and 8Mb.
- P/LP CNVs ≥600Kb
- P/LP CNVs >8Mb.
  - 1. *Aneuploidy Analysis*

The aneuploidy panel included the following specific chromosomal abnormalities:

- Common autosomal trisomies (CAT): Trisomy 21, Trisomy 18, and Trisomy 13
- Sex chromosome aneuploidies (SCA): Monosomy X, Trisomy XXX, Trisomy XYY, and Trisomy XXY
- Rare autosomal aneuploidies (RAA): all autosomal aneuploidies involving chromosomes 1 through 22, excluding CAT. Of note, performance for RAA were calculated on the total samples with prenatal diagnosis on amniotic fluid only.
  1. Fetal cell isolation

To evaluate the performance of the technical workflow, we established two primary metrics. The 'recovery rate' was defined as the proportion of women with at least one putative cEVT recovered at cell sorting, while the 'reportable rate' represented the proportion of women with a confirmed cEVT that produced a usable sequencing profile. For the rate calculations, sample draw with the highest number of putative cEVTs was considered. In cases where both samples gave zero putative cEVTs, the sample collected at the earlier gestational age was used for analysis. In the analyses pertaining to absolute cell counts, only samples with a minimum of one “putative” or “usable” cEVT were included. This exclusion criterion was adopted as the proportion of samples with zero cells was presented separately through the Recovery and Reportable rate assessments.

Technical workflow performance was calculated for the 1360 subjects with a successfully processed scsbNIPT sample.

- 1. Definition of outcomes, exposures, predictors, potential confounders, effect modifiers, diagnostic criteria and missing data

*Outcomes of the study (after mid-study bioinformatic analysis pipeline improvements):* Primary outcome is the performance of scsbNIPT in detecting fetal P/LP CNVs, particularly micro-imbalances <8Mb. This is primarily assessed by comparing scsbNIPT results to those of invasive prenatal diagnosis (CVS or amniocentesis). Secondary outcomes included common aneuploidies, fetal sex, and technology efficiency with cEVTs isolation. We have defined the primary and secondary outcomes in the Methods section, focusing on the concordance between scsbNIPT and the gold standard diagnostic tests.

*Exposures/Predictors:* The "exposure" or predictor is the scsbNIPT test itself. The results of the scsbNIPT are used to predict the presence or absence of fetal chromosomal abnormalities. We also explore factors that might predict the success of obtaining usable cells for the scsbNIPT test. The predictors of obtaining usable cells (gestational age, twin pregnancy) are defined in the Results section and linked to the multivariate logistic regression analysis in the Supplementary Appendix.

*Potential Confounders/Effect Modifiers:* We identified the heterogeneity of criteria adopted across different enrolling sites for pathogenicity classifications in standard diagnostic tests as the main confounding factor in this study that might affect the accuracy of scsbNIPT. To mitigate its effect in the evaluation of the test performances, we implemented a centralized, blinded review of CNV calls to standardize their classification as P/LP rather than benign or VOUS.

*Diagnostic Criteria:*

- *For the reference standard*: karyotyping and CMA, following standard national guidelines (references to national guidelines are provided).
- *For the scsbNIPT test*: we utilized a proprietary algorithm to consolidate results from multiple individual cEVTs, generating a unique subject-level (pregnancy-level) outcome. We also describe the hierarchical priority algorithm used for classifying samples as true positive (TP), false positive (FP), true negative (TN), or false negative (FN), and detail the concordance adjudication process (Fig S1).

*Missing data:* we have described cases not meeting the inclusion criteria, those for which a prenatal diagnosis or a scsbNIPT result could not be obtained for comparison, the adopted strategy of blood redraw and how and why we come up with the final number of 995 cases included in the primary analysis cohort (Fig 1, Supplementary Results and Fig S2). The exclusion of samples without a reportable result means that our reported performance metrics reflect the accuracy of scsbNIPT when a result is achieved. While the reportable rate is an important consideration for clinical implementation, it does not directly impact the validity of the sensitivity and specificity calculations for the successfully analyzed cohort, which is the primary outcome of the study.

1. **Appendix S3 Supplementary results**
   1. Breakdown of sample handling, redraw procedures, and the constitution of the primary analysis cohort

The explanation of the criteria for redraws, the outcomes of redraw, and the reasons for sample exclusion and inclusion with specific numbers and percentages for each step are described below.

In this study, a sample redraw was planned if no putative cEVTs were identified after sorting (protocol version 1) or if fewer than two cEVTs in singleton pregnancies or three in twin pregnancies were isolated (protocol version 2). Consequently, a redraw was requested for 273 of the 1360 samples processed for scsbNIPT (Fig S2).

- *Samples Without Redraw Requests:* a redraw was not requested in 1087 cases, and usable cEVTs were obtained in 928 (85.4%) of these.
- *Outcomes of Redraw Attempts:* a second blood sample was not obtained for 174 of these 273 cases (63.7%). Among the 99 cases where a redraw was obtained (on average 18.5 days later), 56 (56.6%) yielded usable cEVTs. Two of these were excluded because the redraw was performed at >22 weeks of gestation and prenatal diagnostic results were unavailable (e.g: failed analysis or cell culture) for comparison. Therefore, 54 resampled cases were included in the primary analysis cohort.
- *Cases Without scsbNIPT Results:* a scsbNIPT result could not be obtained in 336 cases. This was due to: (1) no resampling being available and no usable cEVTs from the initial sample (n=139); (2) a redraw being available but no usable cEVTs obtained from either the first or second sample (n=43); or (3) putative cEVTs being identified at sorting from the first sample but deemed unusable after genetic analysis (n=159). Of the 341 cases without scsbNIPT results, 5 were excluded due to missing prenatal diagnosis results, leaving 336 cases without usable cEVTs. The reasons for the absence of usable cEVTs were: no putative cEVTs isolated (n=110, 32.7%) or putative cEVTs of insufficient quality (apoptotic, low-quality samples) after isolation (n=226, 67.3%). Multivariate logistic regression analysis comparing the demographics of these 336 pregnancies with the 995 pregnancies with usable fetal cells — including maternal age, weight, height, body-mass index (BMI), gestational week, pregnancy type (singleton vs. twin), and indication for invasive prenatal diagnosis — identified early gestational age and twin pregnancy as the only partial predictors of belonging to the 995 population (Fig S9). The analysis showed that the odds of belonging to the group where usable cEVTs were isolated decreased by 14% [95% CI: 10-15%] for each unit increase in gestational week, while the odds of obtaining usable cEVTs increased by 135% [95% CI: 21-358%] in twin pregnancies. Table S2 reports abnormal CMA findings in these 336 cases.
- *Overall scsbNIPT Results and Primary Analysis Cohort:* overall, a scsbNIPT result was obtained in 1019 cases. Twenty-four of these were excluded due to the absence of prenatal diagnostic results for comparison, resulting in a primary analysis cohort of 995 cases, of whom 54 were resamplings and 57.3% (570/995; one subject was excluded because usable cells were obtained from two samples straddling the 14-week gestational age cutoff for the first trimester) enrolled in the first trimester (Tab S1 and Fig S3–S5). The distribution of first samplings and resamplings across each gestational week is reported in Figure S3. For 11–14 gestational week samples, the majority of them were first samplings and testing on resamplings increased at gestational weeks ≥15, with the highest proportion observed at ≥19 weeks of gestation. Figure S4 presents the proportion of first sampling cases per gestational week for which resampling was requested. Figure S5 illustrates, for the 54 cases where a blood redraw was obtained, the proportion of samples at the gestational age of the first sampling and at the gestational week of the corresponding resampling. Among the 11-14 weeks cases in which early resampling was obtained (16/54; 29.6%), only 31.3% (5/16) of these were delivered to the laboratory in a timely manner (by 15 weeks).

1. **Figures**
   1. Figure S1


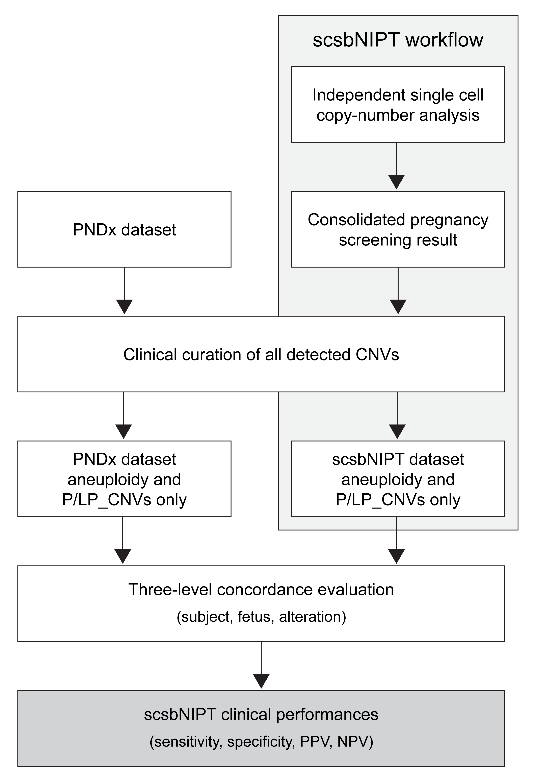


Figure S1: Schematic overview of clinical performance evaluation, based on comparison between single-cell-sequencing-based non-invasive prenatal testing and diagnostic gold standard.

- 1. Figure S2


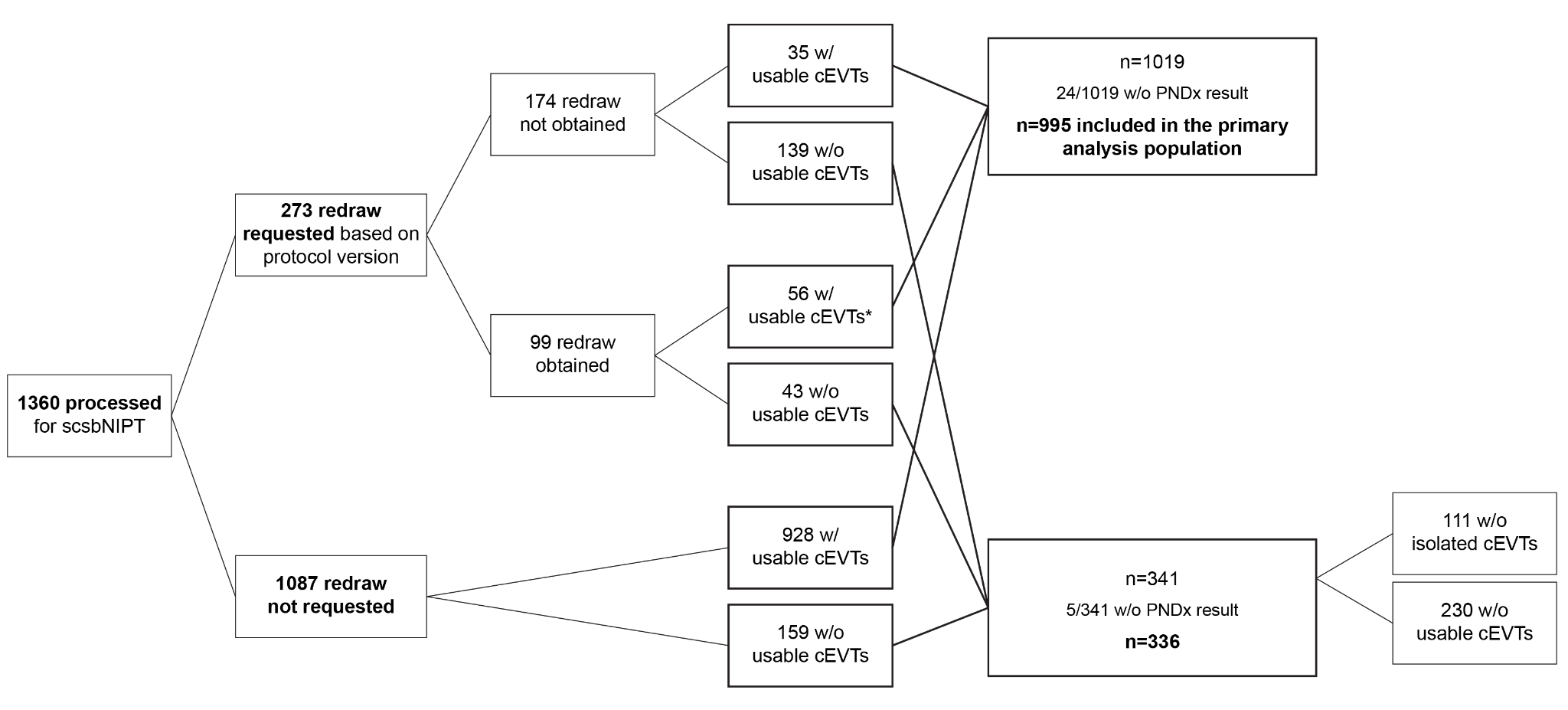


Figure S2: Detailed breakdown of sample handling, redraw procedures and constitution of primary analysis cohort.

Legend: cEVTS, circulating extravillous trophoblasts; PNDx, PreNatal Diagnosis; w/= with; w/o= without; *including cases for which a redraw was requested but 1 or 2 usable cEVTs (based on study protocol v2) were available from the initial sample. Two of these were excluded because the redraw was performed at >22 weeks of gestation and prenatal diagnostic results were unavailable, therefore 54 cases were considered.

- 1. Figure S3

**
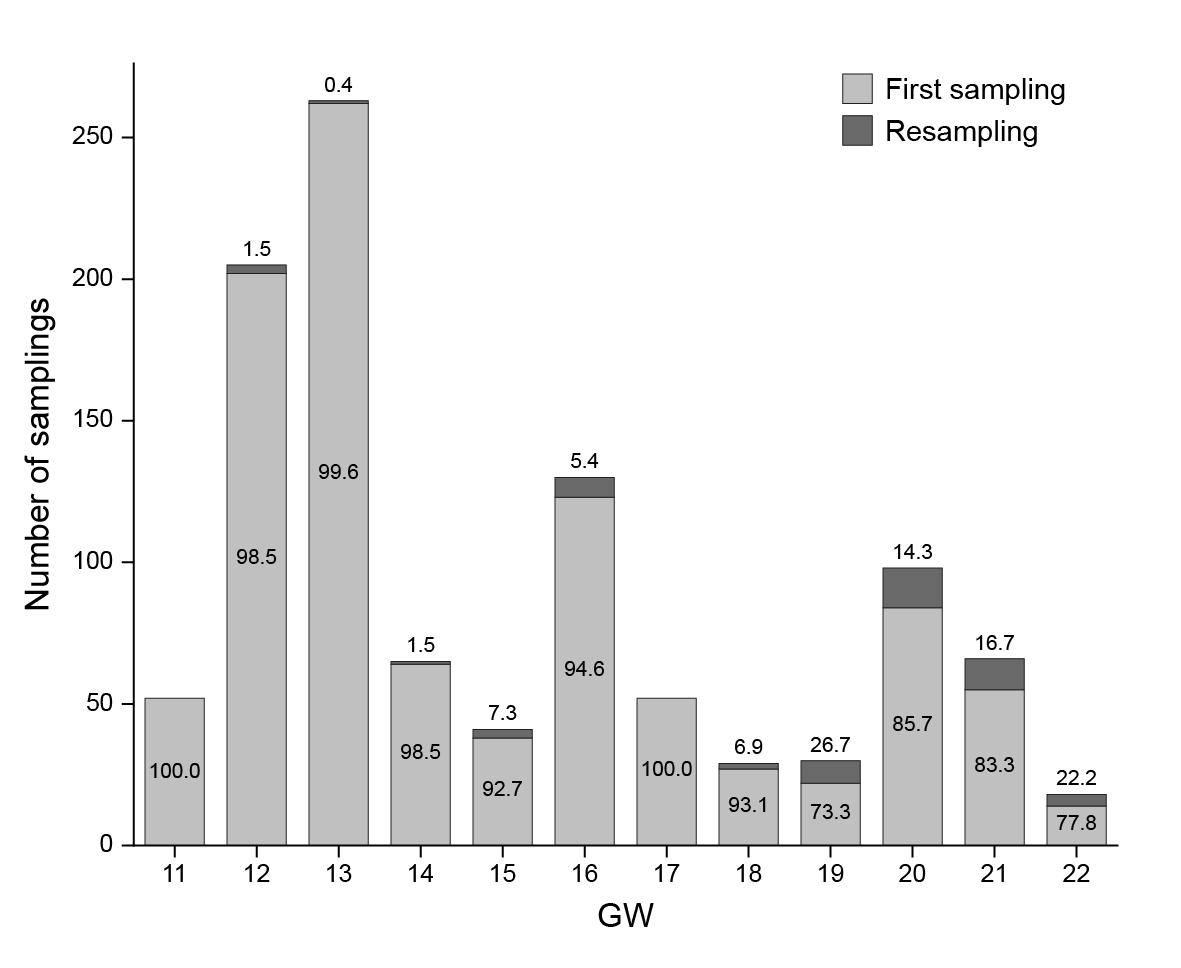
**

Figure S3: Distribution of first blood samplings and obtained redraws (n = 99) in overall cohort (n = 1360), according to gestational week (GW) at collection (numbers represent percentages of total).

- 1. Figure S4


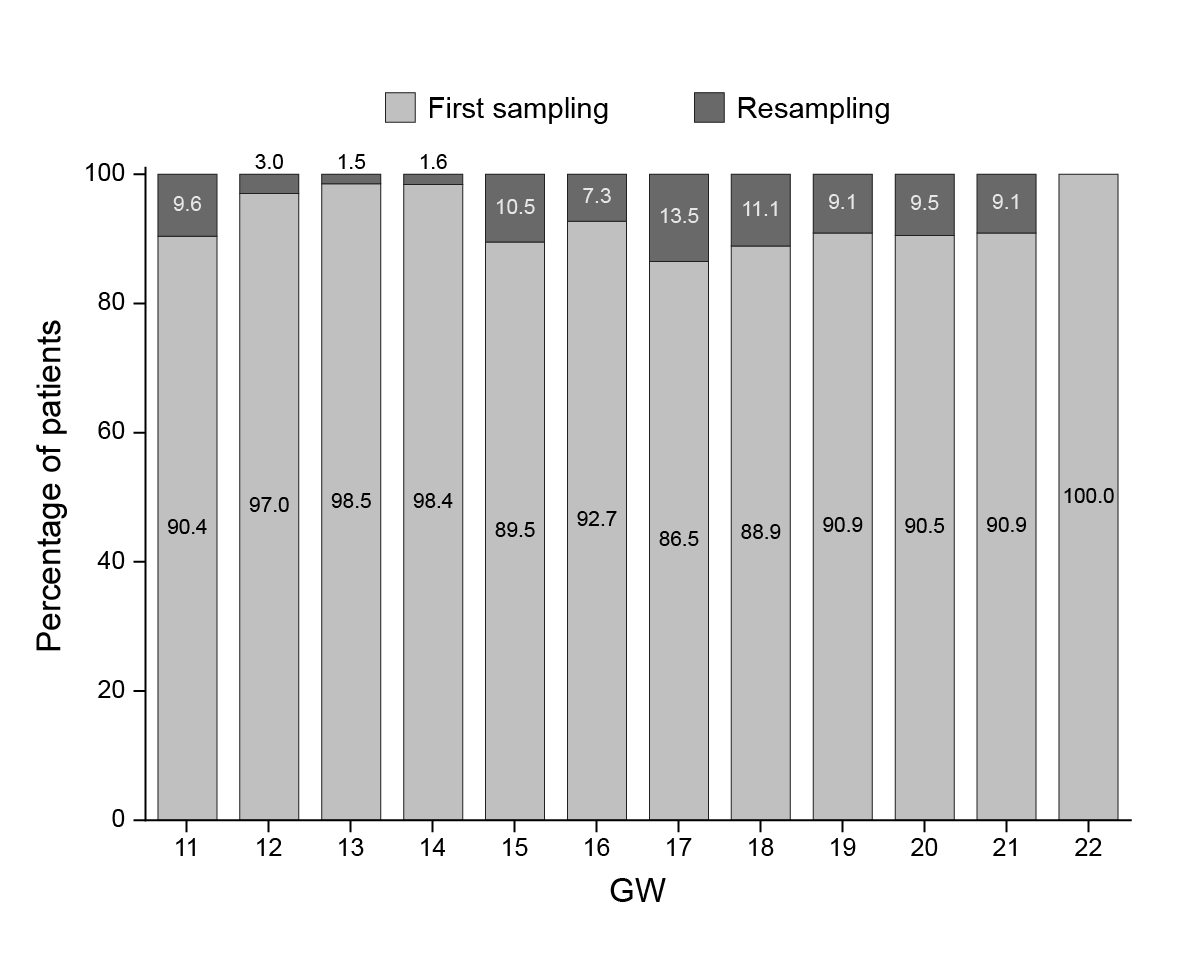


Figure S4: Percentage distribution of first blood samplings and requested redraws in primary analysis cohort (n = 995), according to gestational week (GW) at collection. Note that the resampling fraction (dark grey) represents the fraction of first samplings for which a redraw was obtained (n=54).

- 1. Figure S5

**
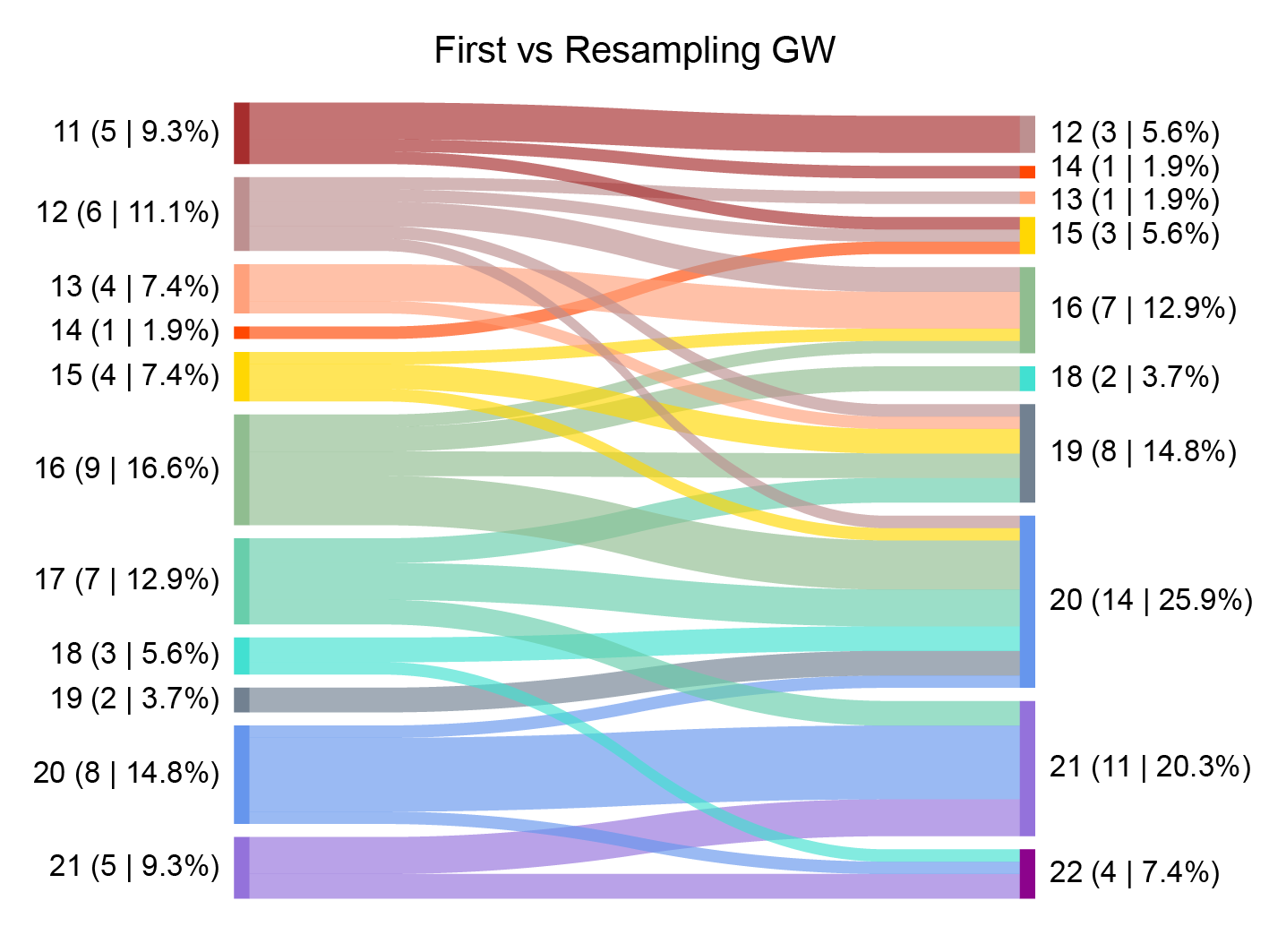
**

Figure S5: Number and proportion of cases by gestational week (GW) for first blood sampling (left) and corresponding resampling (right) in 54 cases for which redraw was obtained and usable circulating extravillous trophoblasts were isolated.

- 1. Figure S6


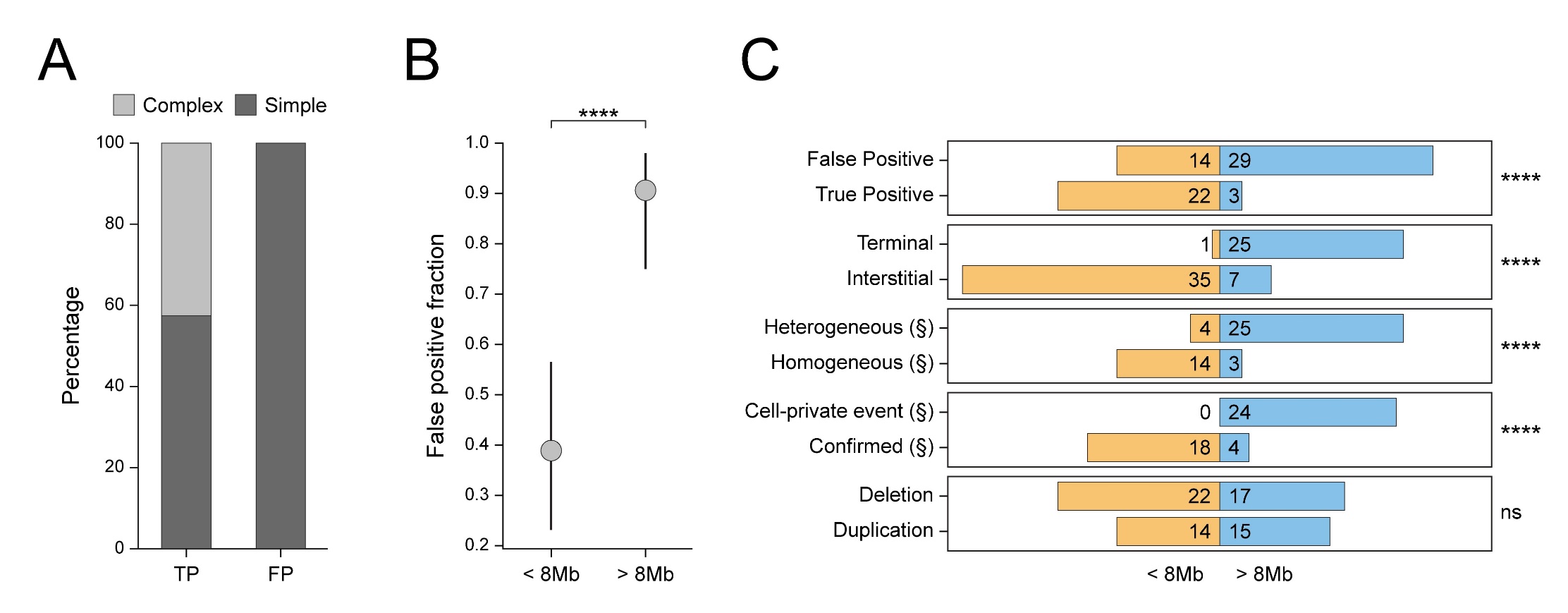


Figure S6. Characterization of pathogenic/likely pathogenic copy-number variants detected by single-cell-sequencing-based non-invasive prenatal testing at alteration level, based on underlying mechanism and cytogenetic features. (**A**) Distribution of one-chromosome ('simple') versus two-chromosome ('complex') imbalances, according to their confirmation outcome (True Positive, TP; or False Positive, FP). All 'complex' imbalances (e.g.: suggestive of a derivative of a parental balanced translocation or a supernumerary marker chromosomes due to an inter-chromosomal rearrangement) were confirmed as TPs. In contrast, 'simple' imbalances were both TPs and FPs. (**B**) Association between one-chromosome imbalance size and FP outcome. The analysis shows a significantly higher probability of a FP result for large imbalances (>8Mb). (**C**) Characterization of the one-chromosome imbalances by size and association with cytogenetic features, namely cell-distribution of the imbalance (homogeneous or heterogeneous/mosaic; in heterogeneous distribution, if present as a cell-private event or in multiple cells), chromosome location (terminal or interstitial), and imbalance type (deletions or duplications). Each box graphically represents the contingency table for each tested pair of conditions, with absolute event counts shown. The key findings are i) a strong association between size, chromosomal location, and cell-distribution so that large imbalances, which are more likely to be false positives, are significantly more prone to be terminal, mosaic and cell-private events while micro-imbalances are more likely to be true positives, interstitial and homogeneous; ii) no relationship was observed between imbalance size and whether the event was a deletion or duplication. Statistically significant associations (p < 0.05, Fisher's Exact test) are marked with asterisks (*), with the number of asterisks proportional to the significance level. All numbers refer to individual alterations, not subjects; therefore, absolute counts may differ from those reported in the main text. (§) To ensure an accurate calculation of the heterogeneity feature, this analysis was restricted to the dataset of alterations from fetuses with at least two cells available for calling.

- 1. Figure S7


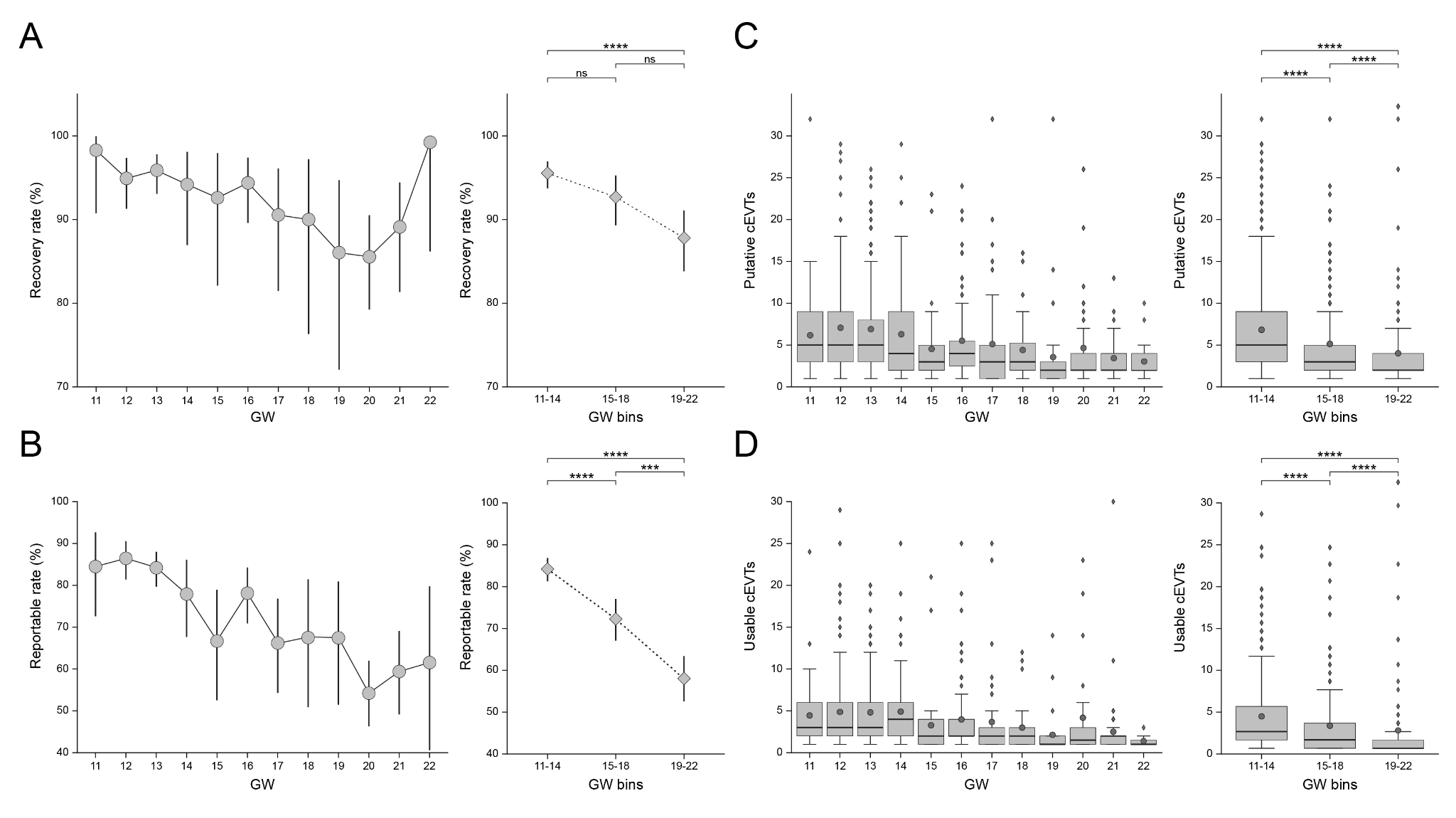


Figure S7. Recovery rate, reportable rate and absolute numbers of putative and usable circulating extravillous trophoblasts isolated per subject in overall cohort (n = 1360), according to gestational age at sample collection. Each subfigure (**A, B, C** and **D**) contains a right and a left panel. The left panel displays the metric under examination (y-axis) for individual gestational weeks (x-axis). The right panel displays the same metric but with gestational weeks grouped into bins: 11-14, 15-18, and 19-22. In the subfigures A and B, the 95% confidence intervals (Clopper-Pearson) are shown for each data point. **A**: a significant decreasing trend in recovery rate was observed (p < 0.0001, Cochran-Armitage Test), with a statistically significant difference identified between the 11-14 week and 19-22 week gestational groups (p < 0.0001, one-sided Fisher’s Exact Test). **B**: a significant decreasing trend in reportable rate was observed (p < 0.0001, Cochran-Armitage Test), with a statistically significant difference identified among all gestational groups (p < 0.001, one-sided Fisher’s Exact Test). The observed decreasing trend is further supported by the absolute counts of both (**C**) putative and (**D**) usable cells, where significant differences were found across all gestational week bins (p<0.0001, Jonckheere-Terpstra; p<0.0001, Kruskal-Wallis with post hoc Dunn’s test). In C and D, only samples with ≥1 cells are included. Overall, the panels illustrate a sharp decrease in the reportable rate, as well as in the counts of both putative and usable cells, starting at 15 weeks' gestation. GW, gestational week.

- 1. Figure S8


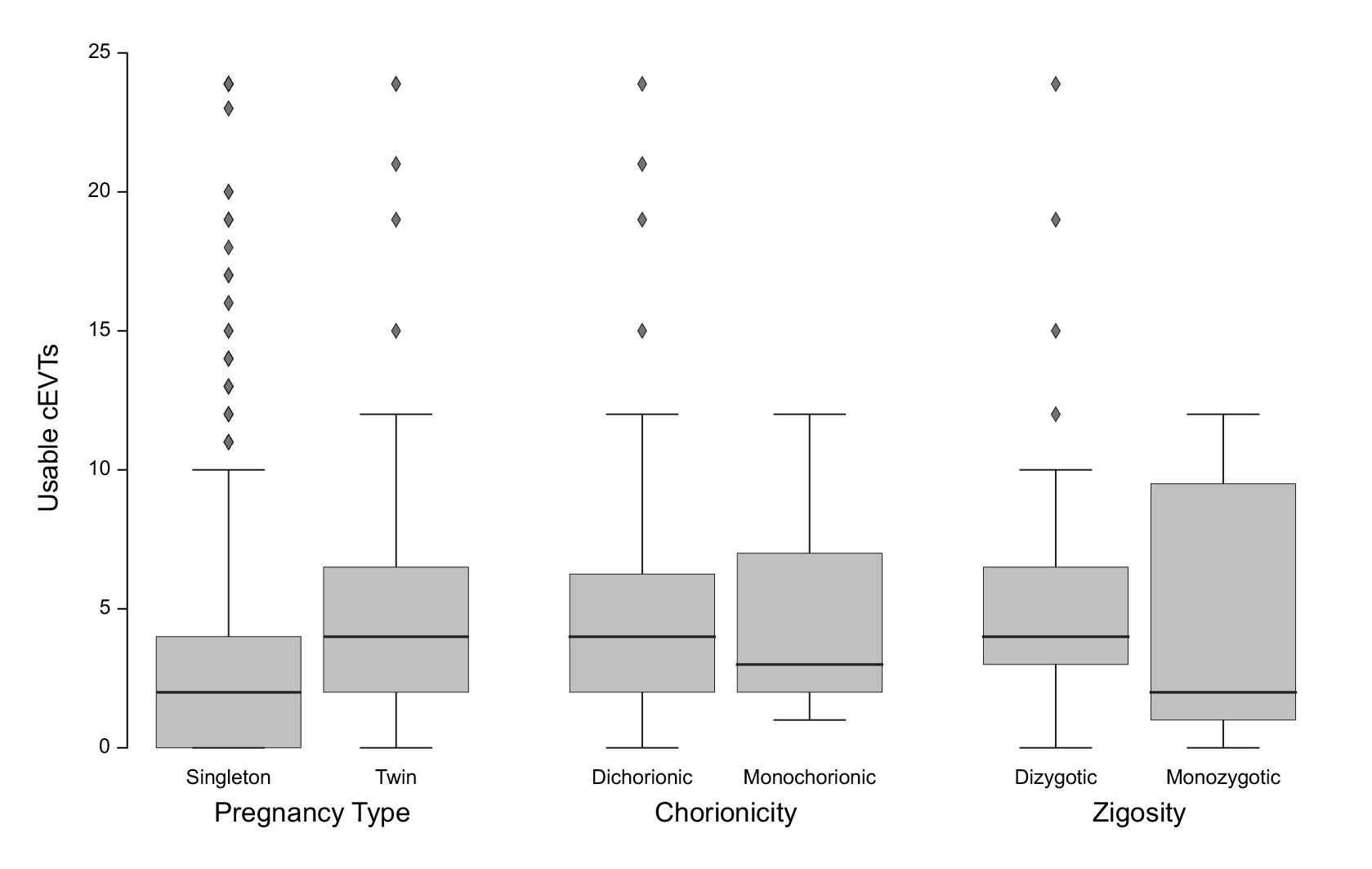


Figure S8: Absolute number of usable circulating extravillous trophoblasts isolated per subject, according to pregnancy type (singleton/twin) and chorionicity and zygosity in twin pregnancies. An analysis of usable cEVTs revealed a significantly greater number in twin pregnancies (average 6.3, median 4) than in singleton pregnancies (average 4.1, median 2) (p < 0.0001, Mann-Whitney-Wilcoxon Test). No significant differences were observed based on chorionicity or zygosity. Similarly, putative cell counts (data not shown) were higher in twin pregnancies (average 9.1, median 6) than in singleton pregnancies (average 5.5, median 4).

- 1. Figure S9


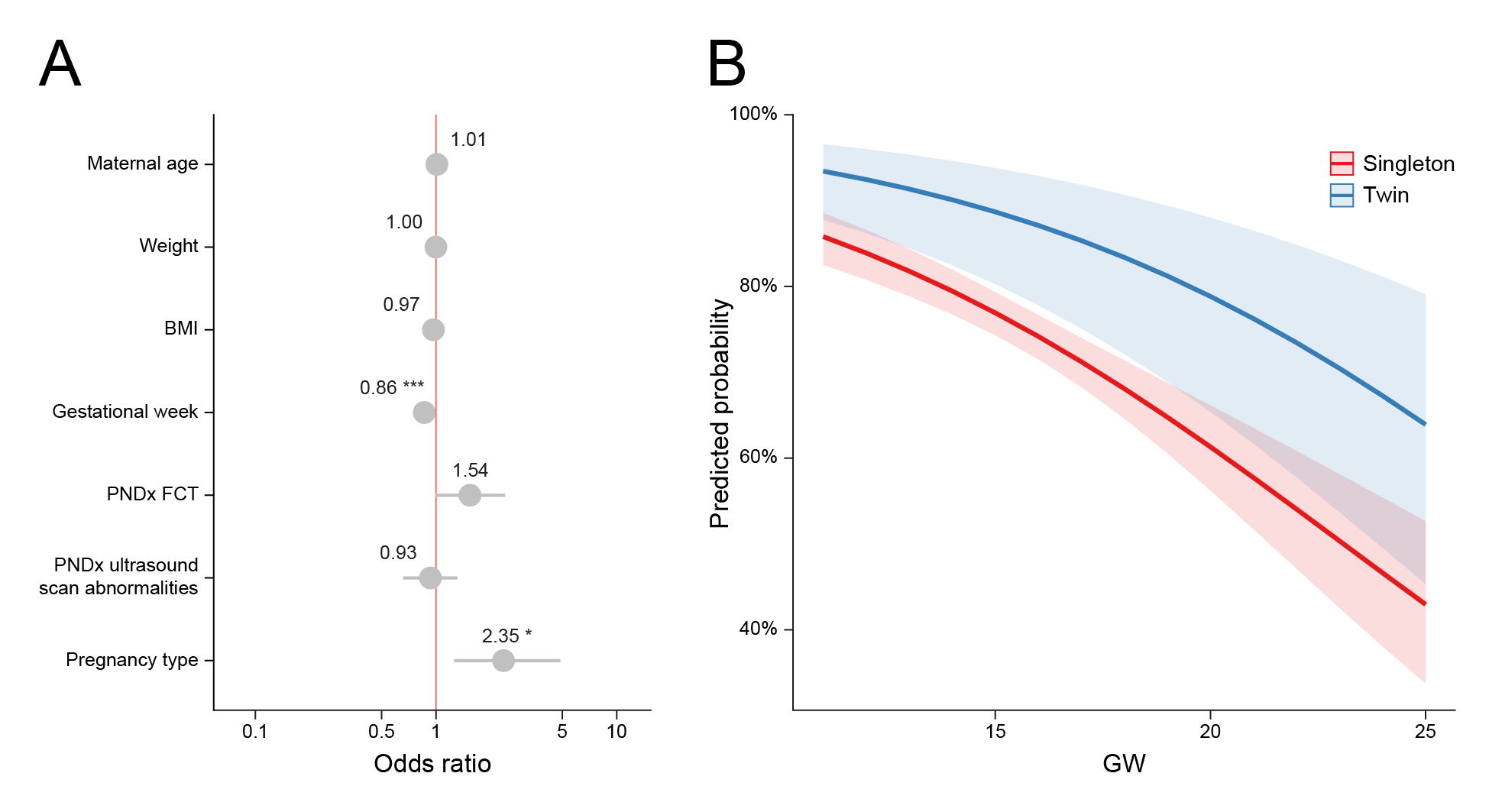


Figure S9. Independent predictors of obtaining usable circulating extravillous trophoblasts. **A:** Forest plot displaying the odds ratios and 95% confidence intervals of demographic factors influencing the acquisition of usable cEVTs (abbreviations: BMI: body mass index, PNDx: prenatal diagnosis, FCT: first‐trimester combined testing; GW, gestational week). Pregnancy type means singleton or twin. **B:** Predicted marginal effects: probability of obtaining usable cEVT as a function of gestational weeks and pregnancy type. Multivariate logistic regression analysis identified twin pregnancy and gestational week as independent predictors of obtaining usable cells.

1. **Tables**
   1. Table S1

| **Table S1:**  **Gestational age distribution of subjects included in primary analysis cohort (n = 995). Fifty-four of them are resamplings.** | |
| --- | --- |
| Gestational week | Subjects — no. (%) |
| 11+0 - 11+6 | 49 (4.92) |
| 12+0 - 12+6 | 198 (19.90) |
| 13+0 - 13+6 | 260 (26.13) |
| 14+0 - 14+6 | 64 (6.43) |
| 15+0 - 15+6 | 36 (3.62) |
| 16+0 - 16+6 | 122 (12.26) |
| 17+0 - 17+6 | 47 (4.72) |
| 18+0 - 18+6 | 27 (2.71) |
| 19+0 - 19+6 | 28 (2.81) |
| 20+0 - 20+6 | 88 (8.84) |
| 21+0 - 21+6 | 60 (6.03) |
| 22+0 - 22+6 | 16 (1.61) |

- 1. Table S2

| **Table S2: List and details of all genomic imbalances detected in screen-positive cases (true and false positives) and screen-negative cases (false negatives) for pathogenic/likely pathogenic copy-number variants in 995 cases included in primary analysis and in 336 cases without result from single-cell-sequencing-based non-invasive prenatal testing** | | | | | | | | | | | |
| --- | --- | --- | --- | --- | --- | --- | --- | --- | --- | --- | --- |
| **CNV ID^** | **Imbalance coordinates (hg19)** | **Early-onset (EO)/Late-onset (LO)/Susceptibility (S)/Variable Penetrance (VP)** | **CNV size (Mb)** | **Gain (G) / Loss (L)** | **Terminal (T)/Interstitial (I)** | **n° abnormal cEVTs/total usable** | **Prenatal Diagnosis details** | | | | |
|  |  |  |  |  |  |  | **Fetal Sex** | **Tissue(s) where the alteration was detected**** | **Assay(s) detecting the alteration***** | **All Tissues analyzed**** | **All Assays performed***** |
| **True positives** | | | | | | | | | | | |
| #001-a | **4q31.3q35.2(152092541_190767114)x1** | EO | 38,67 | L | T | 2/2 | M | CM | CMA | C, CM, M | CMA, K |
| #002 | 22q11.21(20754422_21440514)x1 | EO, VP | 0,69 | L | I | 3/3 | M | CM | CMA | C, CM, M | CMA, K |
| #003 | 22q11.21(18919942_21440514)x1 | EO | 2,52 | L | I | 1/1 | M | AF | CMA | AF | CMA, K |
| #004 | 1p22.3p22.1(87797897_92789142)x1 | EO | 4,99 | L | I | 3/3 | M | CM | CMA | C, CM, M | CMA, K |
| #005-a | 3q27.3q29(186394734_197840339)x3 | EO | 11,45 | G | T | 1/1 | F | C, M | CMA, K | C, M | CMA, K |
| #005-b | 10p15.3p14(148206_11722266)x1 | EO | 11,57 | L | T | 1/1 | F | C, M | CMA, K | C, M | CMA, K |
| #006-a | 8p23.3p21.2(221611_24351346)x3 | EO | 24,13 | G | T | 1/1 | F | C, CM, M | CMA, K | C, CM, M | CMA, K |
| #006-b | 10q26.3(132190695_135404523)x1 | EO | 3,21 | L | T | 1/1 | F | CM | CMA | C, CM, M | CMA, K |
| #007-a | **12p13.33p11.1(230421_34345585)x3** | EO | 34,12 | G | T | 1/1 | F | AF | CMA, K | AF | CMA, K |
| #007-b | **18p11.32(14316_2840500)x1** | EO | 2,83 | L | T | 1/1 | F | AF | CMA | AF | CMA, K |
| #008-a | 7q35q36.3(146236230_158909738)x1 | EO | 12,67 | L | T | 4/4 | M | AF | CMA | AF | CMA |
| #008-b | 8q24.13q24.3(124857091_146280020)x3 | EO | 21,42 | G | T | 4/4 | M | AF | CMA | AF | CMA |
| #009-a | *2q37.3q37.3(237292650_242624663)x1* | EO | 5,33 | L | T | 5/5 | F | C, M | K | C, M | K |
| #009-b | *15q24.1q26.3(72669258_102197282)x3* | EO | 29,53 | G | T | 5/5 | F | C, M | K | C, M | K |
| #010-a | 16p13.3p12.1(106271_26166099)x3 | EO | 26,06 | G | T | 7/7 | M | AF | CMA, K | AF | CMA, K |
| #010-b | 21q22.3(42717594_48067924)x1 | EO | 5,35 | L | T | 7/7 | M | AF | CMA | AF | CMA, K |
| #011 | 13q33.1q34(103100000_115100000)x1 | EO | 12,00 | L | T | 2/2 | M | C, M | K | C, M | K |
| #012 | 1q21.1q21.2(146564743_147786706)x1 | EO, VP | 1,22 | L | I | 2/2 | M | M | CMA | C, M | CMA, K |
| #013-a | 8p23.3p21.2(0_26419091)x3 | EO | 26,42 | G | T | 1/1 | M | CM | CMA | CM | CMA |
| #013-b | 5p15.33p14.1(0_27594662)x1 | EO | 27,59 | L | T | 1/1 | M | CM | CMA | CM | CMA |
| #014 | 1q21.1q21.2(146078110_147869017)x1 | EO, VP | 1,79 | L | I | 40/41 | F | AF | CMA | AF | CMA |
| #015 | 22q11.21(18874332_21490543)x1 | EO | 2,62 | L | I | 1/1 | M | AF | CMA | AF | CMA |
| #016 | 1p36.32(2310193_4632046)x1 | EO | 2,32 | L | I | 3/3 | F | CM | CMA | CM | CMA |
| #017 | 22q11.21(20725987_21474530)x1 mat | EO, VP | 0,75 | L | I | 4/4 | M | AF | CMA | AF | CMA |
| #018 | 16p13.11(14953992_16373837)x1 | S, VP | 1,42 | L | I | 1/1 | F | AF | CMA | AF | CMA |
| #019 | 17q12(34808910_36452972)x3 mat | S, VP | 1,64 | G | I | 1/1 | F | AF | CMA | AF | CMA |
| #020 | 8p23.1(7151932_11880695)x3 mat | EO, VP | 4,73 | G | I | 2/2 | F | CM | CMA | CM | CMA |
| #021 | 13q34(112928717_115169878)x1 | EO | 2,24 | L | T | 2/2 | F | AF | CMA | AF | CMA |
| #022 | 2q11.1q11.2(96774874_98197635)x3 pat | LO**, VP | 1,42 | G | I | 1/1 | F | AF | CMA | AF | CMA |
| #023 | 16p11.2(29673954_30198600)x3 pat | S, VP | 0,52 | G | I | 6/10 | M | CM | CMA | CM | CMA, K |
| #024-a | 18p11.32p11.21(1_15340479)x1 | EO | 15,34 | L | T | 1/1 | F | C, M | K | C, M | K |
| #024-b | 18q11.2q23(19030878_77932526)x3 | EO | 58,90 | G | T | 1/1 | F | C, M | K | C, M | K |
| #025 | 2p24.2p16.3(18295014_50866973)x3 | EO | 32,57 | G | I | 4/4 | M | AF | CMA | AF | CMA |
| #026 | 1q23.1(158390839_158885029)x1 | EO, S | 0,49 | L | I | 1/1 | M | CM | CMA | CM | CMA |
| #027 | 22q11.21(18965499_21086225)x1 | EO | 2,49 | L | I | 10/10 | M | CM | CMA | CM | CMA |
| #028 | 22q11.21(18965499_21086225)x1 | EO | 2,49 | L | I | 9/9 | M | AF | CMA | AF | CMA |
| #029 | Xp11.3(44831882_45185622)amp | EO | 0,35 | G | I | 5/5 | M | CM | CMA | CM | CMA |
| #030-a | 21q11.2q21.3(13268071_27956667)x3 | EO | 14,69 | G | I | 1/1 | M | AF | CMA | AF | CMA |
| #030-b | 2p25.3p24.3(42444_16228425)x3 | EO | 16,37 | G | T | 1/1 | M | AF | CMA | AF | CMA |
| #031-a | 1q42.12q44(225664901_249212666)x3 | EO | 23,55 | G | T | 6/6 | M | C, CM | CMA, K | C, CM | CMA, K |
| #031-b | 11q24.2q25(126564741_134945234)x1 | LO**, VP | 8,38 | L | T | 6/6 | M | C, CM | CMA, K | C, CM | CMA, K |
| #032 | 22q11.21(18890162_20285090)x3 | EO | 1,39 | G | I | 4/4 | M | CM | CMA | C, CM | CMA, K |
| #033 | 16p11.2(29656657_30197466)x3 | S, VP | 0,54 | G | I | 1/1 | M | CM | CMA | CM, M | CMA, K |
| #034-a | 5p15.33p15.2(22149_9980282)x1 | EO | 9,96 | L | T | 1/1 | F | C, CM | CMA, K | C, CM | CMA, K |
| #034-b | 9q33.3q34.3(128668171_141012452)x3 | EO | 12,34 | G | T | 1/1 | F | C, CM | CMA, K | C, CM | CMA, K |
| #035 | 10q11.22q11.23(46174463_51895794)x1 | EO | 5,72 | L | I | 1/1 | M | CM | CMA | C, CM, M | CMA, K |
| #036 | 15q13.3(32021733_32510863)x3 | S, VP | 0,49 | G | I | 1/1 | F | CM | CMA | CM, M | CMA, K |
| **False positives** | | | | | | | | | | | |
| #037 | 5q15q35.3(95300000_180700000)x3 | EO | 85,40 | G | T | 1/1 | F | N/A | N/A | AF | K |
| #038 | 2p25.3p13.2(0_72600000)x1 | EO | 72,60 | L | T | 1/4 | F | N/A | N/A | C, M | K |
| #039 | 10q21.1q26.3(54000000_135200000)x3 | EO | 81,20 | G | T | 1/4 | M | N/A | N/A | C, M | CMA, K |
| #040-a | 9q21.11q34.3(71100000_141000000)x1 | EO | 69,90 | L | T | 1/30 | M | N/A | N/A | AF | CMA, K |
| #040-b | 10p15.3q11.21(200000_42500000)x3 | EO | 42,30 | G | T | 1/30 | M | N/A | N/A | AF | CMA, K |
| #041 | 2q21.1q21.1(131500000_131900000)x1 | S, VP | 0,40 | L | I | 3/3 | M | N/A | N/A | C, CM, M | CMA, K |
| #042 | 16p13.3p13.3(700000_1100000)x1 | LO, VP | 0,40 | L | I | 2/34 | M | N/A | N/A | AF | CMA, K |
| #043 | 1q21.1q44(145000000_249200000)x3 | EO | 104,20 | G | T | 1/13 | M | N/A | N/A | C, M | K |
| #044 | 3q26.32q26.32(176500000_177100000)x3 | EO | 0,60 | G | I | 2/7 | F | N/A | N/A | C, CM, M | CMA, K |
| #006-c | 15q11.2q11.2(22800000_23100000)x1 | S, VP | 0,30 | L | I | 1/1 | F | N/A | N/A | C, CM, M | CMA, K |
| #045 | 16p13.11p13.11(15600000_16300000)x3 | S, VP | 0,70 | G | I | 1/1 | F | N/A | N/A | C, AF, M | CMA, K |
| #046 | 1p13.3q21.3(109100000_154200000)x3 | EO | 45,10 | G | I | 1/3 | M | N/A | N/A | C, CM, M | CMA, K |
| #047 | 6q13q27(74900000_170900000)x1 | EO | 96,00 | L | T | 1/6 | M | N/A | N/A | C, M | CMA, K |
| #048 | 2q12.3q37.3(108600000_243000000)x1 | EO | 134,40 | L | T | 1/33 | M | N/A | N/A | AF | CMA |
| #049 | 18p11.32p11.21(200000_11500000)x2 | EO | 11,30 | L | T | 5/72 | M | N/A | N/A | AF | K |
| #050 | 8q22.3q24.3(102700000_146300000)x3 | EO | 43,60 | G | T | 1/2 | F | N/A | N/A | AF | CMA |
| #051 | 1p12q21.3(120400000_152500000)x3 | EO | 32,10 | G | I | 1/14 | M | N/A | N/A | AF | CMA |
| #052 | 14q11.2q11.2(21700000_23600000)x3 | EO | 1,90 | G | I | 1/1 | N/A | N/A | N/A | CM | CMA |
| #053 | 17q12q12(34800000_36200000)x1 | S, VP | 1,40 | L | I | 1/1 | N/A | N/A | N/A | AF | CMA |
| #054 | 22q11.21q11.21(19000000_21400000)x3 | EO, VP | 2,40 | G | I | 4/4 | N/A | N/A | N/A | CM | CMA |
| #055 | 1q22q22(156000000_156400000)x1 | EO | 0,40 | L | I | 1/1 | N/A | N/A | N/A | CM | CMA |
| #056 | 12q13.12q24.33(50900000_133800000)x3 | EO | 82,90 | G | T | 1/1 | M | N/A | N/A | AF | CMA |
| #057 | 17p12p12(14100000_15400000)x3 | LO** | 1,30 | G | I | 2/2 | M | N/A | N/A | AF | CMA |
| #058 | 11q14.1q25(84600000_134900000)x3 | EO | 50,30 | G | T | 1/1 | M | N/A | N/A | AF | CMA |
| #059 | 21q22.12q22.13(37700000_39600000)x1 | EO | 1,90 | L | I | 1/1 | F | N/A | N/A | AF | CMA |
| #060 | 6p12.1q13(53700000_73800000)x1 | EO | 20,10 | L | I | 1/7 | F | N/A | N/A | CM | CMA |
| #061-a | 1p32.3p12(52800000_120500000)x1 | EO | 67,70 | L | I | 1/51 | M | N/A | N/A | AF | CMA |
| #061-b | 17p13.3p11.2(100000_22200000)x1 | EO | 22,10 | L | T | 1/51 | M | N/A | N/A | AF | CMA |
| #062 | 13q21.32q34(68400000_115100000)x1 | EO | 46,70 | L | T | 1/25 | F | N/A | N/A | AF | CMA |
| #063 | 17q11.2q25.3(26400000_81100000)x1 | EO | 54,70 | L | T | 1/1 | F | N/A | N/A | CM | CMA |
| #064 | 8p23.3p12(400000_30100000)x1 | EO | 29,70 | L | T | 1/3 | F | N/A | N/A | AF | CMA |
| #065 | 3q12.1q24(98300000_147900000)x1 | EO | 49,60 | L | I | 1/17 | M | N/A | N/A | AF | CMA |
| #066 | 1q43q44(242900000_246200000)x3 | EO | 3,30 | G | I | 1/1 | F | N/A | N/A | AF | CMA |
| #067 | 5q12.1q12.1(59400000_59800000)x1 | EO | 0,40 | L | I | 1/1 | M | N/A | N/A | AF | CMA, K |
| #068 | 12p13.33p11.21(200000_33300000)x6 | EO | 33,10 | G | T | 1/3 | F | N/A | N/A | AF | CMA |
| #069 | 7q21.11q36.3(84100000_159100000)x3 | EO | 75,00 | G | T | 1/2 | M | N/A | N/A | AF | CMA |
| #070 | Xp22.33p22.13(2700000_18700000)x3 | EO | 16,00 | G | T | 1/7 | F | N/A | N/A | CM, M | CMA, K |
| #071 | 2q11.2q37.3(96800000_243000000)x1 | EO | 146,20 | L | T | 1/14 | F | N/A | N/A | C | K |
| #034-c | 15q25.2q25.2(84200000_85100000)x1 | EO, VP | 0,90 | L | I | 1/1 | F | N/A | N/A | C, CM | CMA, K |
| #072 | 3p26.3p14.1(100000_67700000)x3 | EO | 67,60 | G | T | 1/5 | F | N/A | N/A | C, CM, M | CMA, K |
| #073-a | 3q22.1q29(130000000_197800000)x1 | EO | 67,80 | L | T | 1/3 | F | N/A | N/A | AF | CMA |
| #073-b | 1q21.1q23.1(145000000_158900000)x3 | EO | 13,90 | G | I | 1/3 | F | N/A | N/A | AF | CMA |
| #073-c | 1q23.2q44(159100000_249200000)x1 | EO | 90,10 | L | T | 1/3 | F | N/A | N/A | AF | CMA |
| **False negatives** | | | | | | | | | | | |
| #001-b | **14q21.2q32.33(44093573_107258824)x3****** | EO | 63,17 | G | T | 0/2 | M | CM | CMA | C, CM, M | CMA, K |
| #074 | Xp21.1(31761210_32118489)x1 pat | EO | 0,36 | L | I | 0/1 | F | AF | CMA | AF | CMA |
| #075 | 15q13.3(31972646_32509926)x3 | S, VP | 0,54 | G | I | 0/1 | M | CM | CMA | CM | CMA |
| **Abnormal CMA findings in cases without a scsbNIPT result (n=336)** | | | | | | | | | | | |
| #076 | **11q13q25(63400000_135006516)x3** | EO | 71,61 | G | T | N/A | M | C | K | C, CM, M | CMA, K |
| #077 | 2q35(215572751_216269907)x1 | EO | 0,70 | L | I | N/A | F | AF | CMA | AF | CMA, K |
| #078-a | 18p11.32p11.21(14316_14928854)x1 | EO | 14,91 | L | T | N/A | F | C, CM, M | CMA, K | C, CM, M | CMA, K |
| #078-b | **18q11.1q23(18539853_77982126)x3** | EO | 59,44 | G | T | N/A | F | M | CMA, K | C, CM, M | CMA, K |
| #079 | Xp11.23p11.22(48317353_52693963)x2 | EO | 4,38 | L | I | N/A | M | AF | CMA | AF | CMA, K |
| #080 | 7q36.3(155598068_158909738)x1 | EO | 3,31 | L | T | N/A | F | CM | CMA | C, CM, M | CMA, K |
| #081-a | 20p13(80198_1891749)x1 | EO | 1,81 | L | T | N/A | F | CM | CMA | C, CM | CMA, K |
| #081-b | 20p13p12.2(1966656_9212087)x3 | EO | 7,25 | G | I | N/A | F | CM | CMA | C, CM | CMA, K |
| #081-c | 20p12.1(13909264_17556606)x1 | LO** | 3,65 | L | I | N/A | F | CM | CMA | C, CM | CMA, K |
| #082-a | 14q32.33(105031444_107349540)x3 | EO, VP | 2,32 | G | T | N/A | M | AF | CMA | AF | CMA |
| #082-b | 17p13.3p13.2(0_4822577)x1 | EO | 4,82 | L | T | N/A | M | AF | CMA | AF | CMA |
| #083 | 20q12q13.33(40363317_66210255)x3 | EO | 25,85 | G | T | N/A | F | CM | CMA | CM | CMA |
| #084 | 3q22.3(136215510_136795199)x3 mat | EO, VP | 0,58 | G | I | N/A | F | CM | CMA | CM | CMA |
| #085 | Xp22.33(0_2697868)x3 pat | EO, VP | 2,70 | G | T | N/A | M | AF | CMA | AF | CMA |
| #086 | 22q11.21(20597993_21630770)x1 pat | EO | 1,03 | L | I | N/A | M | AF | CMA | AF | CMA |
| #087 | 15q13.3(32019325_32618963)x1 pat | S, VP | 0,60 | L | I | N/A | F | AF | CMA | AF | CMA |
| #088 | 15q15.3(43851025_43939650)x1 mat | LO, S | 0,09 | L | I | N/A | F | AF | CMA | AF | CMA |
| #089 | 16q22.1q23.2(69748897_81165249)x1 | EO | 11,42 | L | I | N/A | F | CM | CMA, K | CM | CMA, K |
| #090 | 3q29(197574293_197840339)x3 pat | EO, VP | 0,27 | G | T | N/A | N/A | AF | CMA | AF | CMA, K |
| #091 | 8p23.1(11350104_11399826)x1 | LO**, VP | 0,05 | L | I | N/A | M | AF | CMA | AF | CMA |
| #092 | 2q37.3(242886386_243007359)x1 | S, VP | 0,12 | L | T | N/A | M | AF | CMA | AF | CMA |
| #093 | 9p24.3p23(12934_10340841)x1 | EO | 10,33 | L | T | N/A | F | AF | CMA, K | AF | CMA, K |
| #094 | 16p11.2(29656657_30197466)x3 | S, VP | 0,54 | G | I | N/A | F | AF | CMA | AF | CMA, K |
| ^'a', 'b' and 'c' assigned to the same CNV ID indicate CNVs present in the same pregnancy  *Aneuploidies are excluded; N/A, Not Applicable; Mb, Megabase; cEVTs, circulating extravillous trophoblasts; **Simptoms may arise in puberty/adolescence  ** AF = Amniotic Fluid, C = Chorionic villi Cytotrophoblast (direct), M = Chorionic villi Mesenchyme (long term), CM = Chorionic villi Fresh biopsy *** CMA = array-CGH/SNP-array , K = Karyotype **** This alteration was not considered as a false negative because a true positive alteration was concurrently detected and prioritized in the final adjudicated result *(italic) genomic coordinates were derived previous characterization of paternal translocation known before prenatal diagnosis* **(bold) PNDx reported mosaicism for alteration** | | | | | | | | | | | |

- 1. Table S3

| **Table S3: Performance of single-cell-sequencing-based non-invasive prenatal testing for diagnosis of pathogenic/likely pathogenic copy-number variants and common aneuploidies, calculated at fetus level** | | | | | | | | | | | | |
| --- | --- | --- | --- | --- | --- | --- | --- | --- | --- | --- | --- | --- |
| **Variable** | **300Kb-8Mb** | **≥300Kb** | **≥8Mb** | **Trisomy 21** | **Trisomy 18** | **Trisomy 13** | **45,X** | **47,XXX** | **47,XXY** | **47,XYY** | **CAT+SCA+**  **pCNV≥300Kb** | **CAT+SCA+**  **pCNV300Kb-8Mb** |
| True positive — no. | 26 | 36 | 14 | 98 | 19 | 3 | 6 | 3 | 4 | 2 | 169 | 159 |
| True negative — no. | 739 | 964 | 1000 | 935 | 1018 | 1033 | 1027 | 1032 | 1035 | 1035 | 818 | 846 |
| False positive — no. | 13 | 37 | 25 | 3 | 2 | 3 | 4 | 3 | 0 | 0 | 44 | 26 |
| False negative — no. | 2 | 2 | 0 | 3 | 0 | 0 | 2 | 1 | 0 | 2 | 8 | 8 |
| Sensitivity (95% CI) — % | 92.9  (76.5 - 99.1) | 94.7  (82.3 - 99.4) | 100.0  (76.8 - 100.0) | 97.0  (91.6 - 99.4) | 100.0  (82.4 - 100.0) | 100.0  (29.2 - 100.0) | 75.0  (34.9 - 96.8) | 75.0  (19.4 - 99.4) | 100.0  (39.8 - 100.0) | 50.0  (6.8 - 93.2) | 95.5  (91.3 - 98.0) | 95.2  (90.8 - 97.9) |
| Specificity (95% CI) — % | 98.3  (97.1 - 99.1) | 96.3  (94.9 - 97.4) | 97.6  (96.4 - 98.4) | 99.7  (99.1 - 99.9) | 99.8  (99.3 - 100.0) | 99.7  (99.2 - 99.9) | 99.6  (99.0 - 99.9) | 99.7  (99.2 - 99.9) | 100.0  (99.6 - 100.0) | 100.0  (99.6 - 100.0) | 94.9  (93.2 - 96.3) | 97.0  (95.7 - 98.0) |
| Positive predictive value (95% CI) — % | 66.7  (49.8 - 80.9) | 49.3  (37.4 - 61.3) | 35.9  (21.2 - 52.8) | 97.0  (91.6 - 99.4) | 90.5  (69.6 - 98.8) | 50.0  (11.8 - 88.2) | 60.0  (26.2 - 87.8) | 50.0  (11.8 - 88.2) | 100.0  (39.8 - 100.0) | 100.0  (15.8 - 100.0) | 79.3  (73.3 - 84.6) | 85.9  (80.1 - 90.6) |
| Negative predictive value (95% CI) — % | 99.7  (99.0 - 100.0) | 99.8  (99.3 - 100.0) | 100.0  (99.6 - 100.0) | 99.7  (99.1 - 99.9) | 100.0  (99.6 - 100.0) | 100.0  (99.6 - 100.0) | 99.8  (99.3 - 100.0) | 99.9  (99.5 - 100.0) | 100.0  (99.6 - 100.0) | 99.8  (99.3 - 100.0) | 99.0  (98.1 - 99.6) | 99.1  (98.2 - 99.6) |
| Legend: CAT, common autosomal trisomies; SCA, Sex chromosome aneuploidies including 45,X, 47,XXX, 47,XXY and 47,XYY | | | | | | | | | | | | |

- 1. Table S4

| **Table S4: Performance of single-cell-sequencing-based non-invasive prenatal testing for diagnosis of pathogenic/likely pathogenic copy-number variants and common aneuploidies, calculated at alteration level** | | | | | | | | | | | | |
| --- | --- | --- | --- | --- | --- | --- | --- | --- | --- | --- | --- | --- |
| **Variable** | **300Kb-8Mb*** | **≥300Kb*** | **≥8Mb*** | **Trisomy 21** | **Trisomy 18** | **Trisomy 13** | **45,X** | **47,XXX** | **47,XXY** | **47,XYY** | **CAT+SCA+**  **pCNV≥300Kb*** | **CAT+SCA+**  **pCNV300Kb-8Mb*** |
| True positive — no. | 26 | 47 | 21 | 98 | 19 | 3 | 6 | 3 | 4 | 2 | 182 | 161 |
| True negative — no. | na | na | na | 935 | 1018 | 1033 | 1027 | 1032 | 1035 | 1035 | na | na |
| False positive — no. | 14 | 43 | 29 | 3 | 2 | 3 | 4 | 3 | 0 | 0 | 58 | 29 |
| False negative — no. | 2 | 3 | 1 | 3 | 0 | 0 | 2 | 1 | 0 | 2 | 12 | 11 |
| Sensitivity (95% CI) — % | 92.9  (76.5 - 99.1) | 94.0  (83.5 - 98.7) | 95.5  (77.2 - 99.9) | 97.0  (91.6 - 99.4) | 100.0  (82.4 - 100.0) | 100.0  (29.2 - 100.0) | 75.0  (34.9 - 96.8) | 75.0  (19.4 - 99.4) | 100.0  (39.8 - 100.0) | 50.0  (6.8 - 93.2) | 93.8  (89.4 - 96.8) | 93.6  (88.8 - 96.8) |
| Specificity (95% CI) — % | na | na | na | 99.7  (99.1 - 99.9) | 99.8  (99.3 - 100.0) | 99.7  (99.2 - 99.9) | 99.6  (99.0 - 99.9) | 99.7  (99.2 - 99.9) | 100.0  (99.6 - 100.0) | 100.0  (99.6 - 100.0) | na | na |
| Positive predictive value (95% CI) — % | 65.0  (48.3 - 79.4) | 52.2  (41.4 - 62.9) | 42.0  (28.2 - 56.8) | 97.0  (91.6 - 99.4) | 90.5  (69.6 - 98.8) | 50.0  (11.8 - 88.2) | 60.0  (26.2 - 87.8) | 50.0  (11.8 - 88.2) | 100.0  (39.8 - 100.0) | 100.0  (15.8 - 100.0) | 75.8  (69.9 - 81.1) | 84.7  (78.8 - 89.5) |
| Negative predictive value (95% CI) — % | na | na | na | 99.7  (99.1 - 99.9) | 100.0  (99.6 - 100.0) | 100.0  (99.6 - 100.0) | 99.8  (99.3 - 100.0) | 99.9  (99.5 - 100.0) | 100.0  (99.6 - 100.0) | 99.8  (99.3 - 100.0) | na | na |
| Legend: (*) For the categories listed, specificity and NPV could not be calculated. This requires quantifying True Negatives (TN), which depends on an a priori definable number of testable events. For a specific aneuploidy (e.g., T21), this equals the number of fetuses. In contrast, for alteration size ranges, the number of potentially callable, unique events is not predictable. CAT= common autosomal trisomies; SCA= Sex chromosome aneuploidies | | | | | | | | | | | | |

- 1. Table S5

| **Table S5: Performance of single-cell-sequencing-based non-invasive prenatal testing for determination of fetal sex** | | |
| --- | --- | --- |
|  | **fetuses (n)** | **Proportion (%)** |
| Male | 433 | 52.7 |
| Female | 388 | 47.3 |
|  | **Subjects (n)** | **Fetuses (n)** |
| Concordant | 796 | 821 |
| Discordant | 0 | 0 |
| No call | 199 | 243 |
| Total | 995 | 1064 |
| Concordance (95% CI) — % | 100 (99.5-100.0) | 100 (99.5-100.0) |

- 1. Table S6

| **Table S6:**  **Cumulative performance of single-cell-sequencing-based non-invasive prenatal testing for rare autosomal aneuploidies** | |
| --- | --- |
| **Variable** | **Overall RAA (n=442*)** |
| True positive — no. | 4 |
| True negative — no. | 421 |
| False positive — no. | 16 |
| False negative — no. | 1 |
| Sensitivity (95% CI) — % | 80.0 (28.4 - 99.5) |
| Specificity (95% CI) — % | 96.3 (94.1 - 97.9) |
| Positive predictive value (95% CI) — % | 20.0 (5.7 - 43.7) |
| Negative predictive value (95% CI) — % | 99.8 (98.7 - 100.0) |
| *Performance are calculated only on cases with a diagnostic confirmation on amniotic fluid | |

Legend: RAA, Rare Autosomal Aneuploidies

- 1. Table S7

| **Table S7: Recovery rate, reportable rate and absolute numbers of putative and usable circulating extravillous trophoblasts isolated per subject in overall cohort (n = 1360), according to gestational age at sample collection** | | | | | | | | | | | | |
| --- | --- | --- | --- | --- | --- | --- | --- | --- | --- | --- | --- | --- |
| Cell type | GW | total samples (n) | samples without cells (n) | samples with cells (n) | rate | mean | std | min | 25% | 50% | 75% | max |
| putative | 11-14 | 696 | 31 | 665 | 95.5 | 6.8 | 7.1 | 1 | 3 | 5 | 9 | 98 |
| putative | 15-18 | 328 | 24 | 304 | 92.7 | 5.1 | 6.6 | 1 | 2 | 3 | 5 | 65 |
| putative | 19-22 | 336 | 41 | 295 | 87.8 | 4.0 | 7.3 | 1 | 1 | 2 | 4 | 60 |
| usable | 11-14 | 696 | 110 | 586 | 84.2 | 4.8 | 5.5 | 1 | 2 | 3 | 6 | 72 |
| usable | 15-18 | 328 | 91 | 237 | 72.3 | 3.7 | 5.1 | 1 | 1 | 2 | 4 | 53 |
| usable | 19-22 | 336 | 141 | 195 | 58.0 | 3.1 | 6.3 | 1 | 1 | 1 | 2 | 51 |

Legend: GW, gestational week; std, standard deviation

1. **References**
2. [Doffini A, Forcato C, Mangano C, et al. Isolation of single circulating trophoblasts from maternal circulation for noninvasive fetal copy number variant profiling. Prenat Diagn 2023;43(1):14–27.](http://paperpile.com/b/R6tbaU/0zDOA)
3. [Wapner RJ, Martin CL, Levy B, et al. Chromosomal microarray versus karyotyping for prenatal diagnosis. N Engl J Med 2012;367(23):2175–84.](http://paperpile.com/b/R6tbaU/FiUw)
4. de Wit MC, Srebniak MI, Govaerts LC, et al. Additional value of prenatal genomic array testing in fetuses with isolated structural ultrasound abnormalities and a normal karyotype: a systematic review of the literature. Ultrasound Obstet Gynecol. 2014 Feb;43(2):139-46.
5. Grati FR, Bestetti I, De Siero D, et al. Positive predictive values and outcomes for uninformative cell-free DNA tests: An Italian multicentric Cytogenetic and cytogenomic Audit of diagnOstic testing (ICARO study). Prenat Diagn. 2022 Dec;42(13):1575-1586.
6. Gardner M.R.J., Amor D.J. (2018) Chromosome abnormalities detected at prenatal diagnosis. In: Gardner and Sutherland's Chromosome Abnormalities and Genetic Counseling; 5th Edition. Oxford University Press monographs on medical genetics; p466-515)
7. Silva M, de Leeuw N, Mann K, et al. European guidelines for constitutional cytogenomic analysis. Eur J Hum Genet. 2019 Jan;27(1):1-16.
8. McGowan‐Jordan J, Simons A, Schmid M, eds. ISCN 2016 An International System for Human Cytogenomic Nomenclature. Basel: S Karger Ag; 2016.
